# A Novel Prioritization Method in Identifying Recurrent Venous Thromboembolism-Related Genes

**DOI:** 10.1371/journal.pone.0153006

**Published:** 2016-04-06

**Authors:** Jing Jiang, Wan Li, Binhua Liang, Ruiqiang Xie, Binbin Chen, Hao Huang, Yiran Li, Yuehan He, Junjie Lv, Weiming He, Lina Chen

**Affiliations:** 1 College of Bioinformatics Science and Technology, Harbin Medical University, Harbin, Hei Longjiang Province, China, Postal code: 150081; 2 National Microbology Laboratory, Public Health Agency of Canada, Winnipeg, Manitoba, Canada; 3 Institute of Opto-electronics, Harbin Institute of Technology, Harbin, Hei Longjiang Province, China; Huazhong University of Science and Technology, CHINA

## Abstract

Identifying the genes involved in venous thromboembolism (VTE) recurrence is important not only for understanding the pathogenesis but also for discovering the therapeutic targets. We proposed a novel prioritization method called Function-Interaction-Pearson (FIP) by creating gene-disease similarity scores to prioritize candidate genes underling VTE. The scores were calculated by integrating and optimizing three types of resources including gene expression, gene ontology and protein-protein interaction. As a result, 124 out of top 200 prioritized candidate genes had been confirmed in literature, among which there were 34 antithrombotic drug targets. Compared with two well-known gene prioritization tools Endeavour and ToppNet, FIP was shown to have better performance. The approach provides a valuable alternative for drug targets discovery and disease therapy.

## Introduction

Venous thromboembolism (VTE) is the third most common cardiovascular disease with a high risk of recurrence and mortality [1–5]. It was reported that around one-third of patients suffering from a first episode of deep venous thrombosis (DVT) or pulmonary embolism (PE) developed a VTE recurrence within 10 years [6]. Even during warfarin anticoagulant therapy, VTE-experienced patients still face risks of recurrent VTE [7–9]. In clinical practice, it is helpful to identify biomarkers that aid the early diagnosis of patients at a high or low risk of primary and recurrent VTE, and assess therapy [10].

In the past, efforts had been exerted on seeking these biomarker [11]. Through whole blood gene expression analysis, the D-dimer [12], the soluble p-selectin [13], and the thrombin [14] were found to be strongly associated with an increased risk of recurrent VTE and thus were accepted as biomarkers of recurrent VTE [15,16]. However, there were limitations in determining biomarkers of recurrent VTE through whole blood gene expression analysis. At first, the VTE patient population was a heterogeneous mixture of patients with provoked and non-provoked VTEs. Secondly, the two groups of VTE patients differed in the duration of time since their last VTE as well as duration of warfarin therapy. At last, some patients with a single VTE would likely be vulnerable to a recurrent event if anticoagulant therapy discontinued, resulting in reclassification of any affected individual [16]. Differential expression analysis might not determine which genes were more important or could neglect some potential disease-related genes [17].

Alternately, the computational methods such as prioritization methods, including ToppNet (https://toppgene.cchmc.org/network.jsp) [18] and Endeavour (http://homes.esat.kuleuven.be/~biouser/endeavour/tool/endeavourweb.php) [19], were deployed to investigate potential disease genes [20–22]. These methods assume that both the potential disease-related genes and the known genes share functions, interact with each other, and are involved in similar phenotypes. The studied genes were assigned similarity or confidence scores with disease followed by the ranking based on the descending order of the scores. In general, these prioritization methods rely on functional annotations [23,24], network properties [25–28] and gene expression data [29–31]. ToppNet ranks or prioritizes genes based on topological features in the protein-protein interaction network (PPIN). ToppNet has been applied with good performances in a few studies [32–34]. For example, Lascorz et al. applied ToppNet tool in identifying markers of colorectal cancer. The three overrepresenting genes was found to be closely related to the mitogen-activated protein kinase (MAPK) signaling pathways, which is well-known to increase the risk of colorectal cancer [35]. In another study by using ToppNet, the OPRM1 gene was shown to be significantly differently expressed between different HIV groups [36]. The weakness of ToppNet is only one data source used for ranking genes which affects its robustness for candidate gene identification.

Inspirited by the fact that that integrative strategy in combining distinct resources showed a better performance in discovery of disease-related genes [37–46], Endeavour was developed [47]. Endeavour integrates 19 distinct data sources, including annotation (Gene Ontology, Swissprot, Interpro, Kegg, EnsemblEst), Interaction (Bind, String, BioGrid, Hprd, InNetDb, Intact, Mint), Expression (SonEtAl, SuEtAl), Precalculated (Ouzounis, Prospectr), Motif, Blast, and Text mining. The rankings of the candidates derived from each source were further combined into one global ranking. Robert et al. ranked the differentially expressed genes through Endeavour and identified P2rx7 (the 2nd ranked) and P2rx4 (the 3rd ranked) responsible for impaired blood pressure control in rat. The result was confirmed by Western analysis which was consistent with the previous congenic studies [48]. In Kamron et al.’s study, candidate genes of congenital cataract were prioritized using Endeavour and the three top-ranked genes were confirmed to be associated with the disease by literature [49]. The limitation of Endeavour was that it did not take disease samples into account [50]. In fact, the accuracy of prioritization methods is directly correlated with the quality of data [51]. Moreover, Endeavour solely depends on the protein interactions defined in the databases for gene prioritization. However, many protein-protein links in the databases are very loose since structural or chemical properties and functionalities were not taken into consideration, leading to reduced protein interaction reliabilities.

In this study, we present FIP (Function-Interaction-Pearson), a novel prioritization method designed for identifying Recurrent Venous Thromboembolism-related Genes. FIP addressed the limitations of the current commonly used methods in prioritizing genes. Potential VTE recurrence related genes were identified as the top-ranked genes. Our study would provide a valuable alternative for enhancing our understanding of the complex molecular mechanism of VTE recurrence at a system level.

## Materials and Methods

### Data Source

A gene expression profile of whole blood was downloaded from the publicly available Gene Expression Omnibus (GEO, http://www.ncbi.nml.nih.gov/geo/) [52]. The profile GSE19151 in the platform GPL571 was selected for downstream analysis in this study. GSE19151 contains 13785 genes derived from the 133 samples in different groups, including normal subjects (63), single event VTE patients (32), and recurrent VTE patients (38) who are on warfarin therapy. The differentially expressed genes were identified by using the Significance Analysis of Microarrays (SAM) between normal and recurrent samples. The 119 thrombosis disease-related genes were obtained from Online Mendelian Inheritance in Man (OMIM, http://omim.org/) [53], Genetic Association Database (GAD, http://geneticassociationdb.nih.gov/) [54] and Disease Ontology (DO, http://disease-ontology.org/) [55]. The interaction network used in this study was downloaded from STRING (http://string-db.org/) [56]. In the network, gene association datasets were either directly derived from physical interactions or functional links from experimental evidence and computational methods [57,58]. The network composes of 5260 nodes (disease-related genes and differential genes in the interaction network) and 42087 edges, which represent genes and interactions between them, respectively. In our study, 108 disease-related genes (excluded 11 genes not in the STRING database and the profile GSE19151) were selected as seed genes and other genes as candidate genes.

### The FIP method

**Fig 1:**
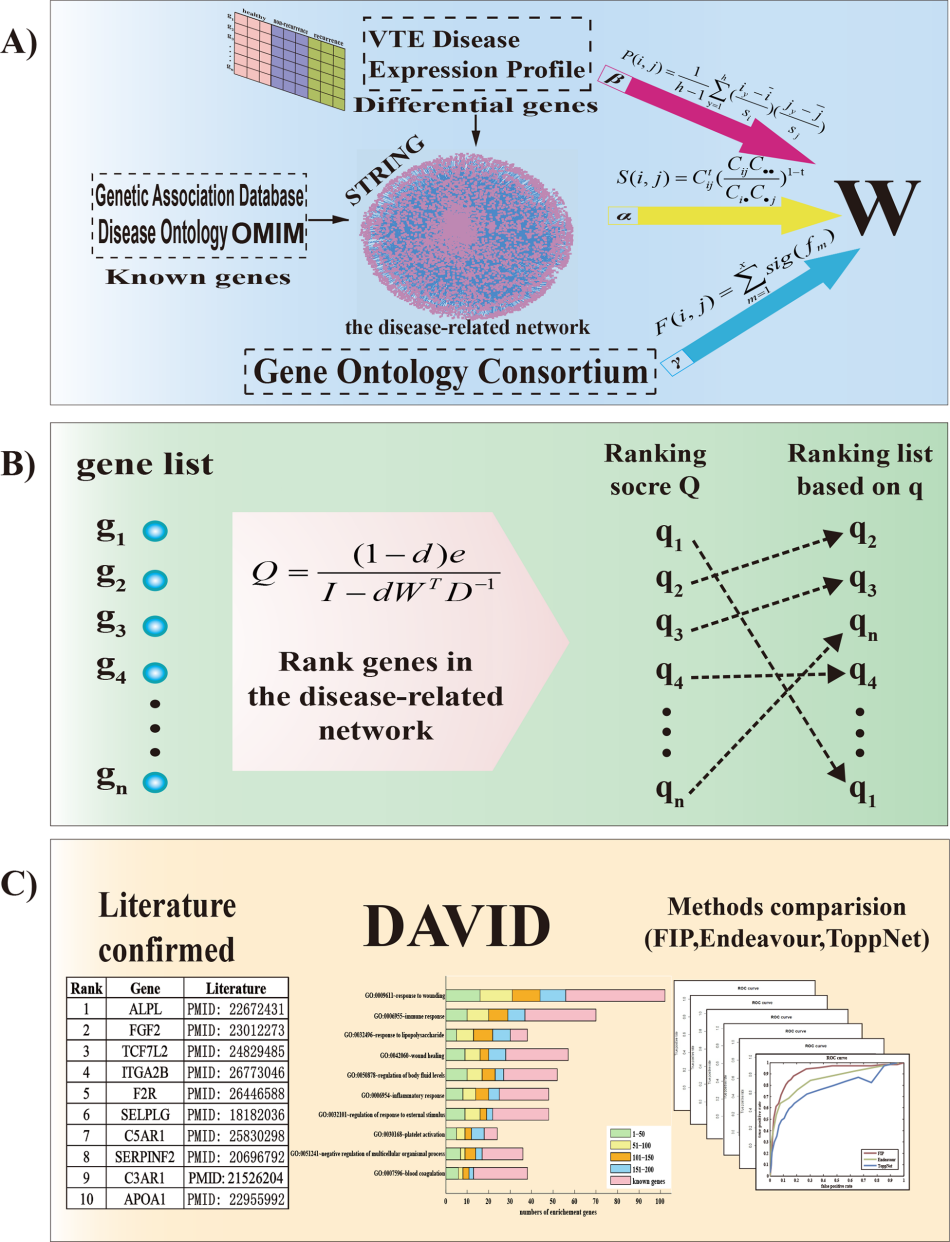
The workflow of FIP method and its validations. A: measurement of the overall similarity between genes. B: calculation of ranking scores of candidate genes. C: verification of the performance of the results.

A novel prioritization method FIP was developed to prioritize VTE candidate genes by calculating gene-disease similarity scores, also called disease relevance score q. Briefly, the disease relevance score for each gene was measured by considering the overall similarity with its neighboring genes in the disease-related network based on the separated data sources: gene ontology, protein-protein interaction, and gene expression. The workflow of the method and its validations were described below (Fig 1).

The score vector Q (n×1; n—the total number of genes) represented disease relevance scores for all genes in the disease-related network, which was formulated as follows:

$$Q=\frac{(1-d)e}{I-dW^{T}D^{-1}}$$ (1)

where q_i_ (q_i_∈Q) is the ranking score of gene i, I denotes an identity matrix of n×n, *e* is the expression score vector of n×1 where *e*_i_ is defined as the absolute value of the difference between the sum of expression values of gene *i* in normal and recurrent samples, *d* denotes a control parameter in the range of [0,1] which is to adjust the weight of disease-related network in calculating ranking scores (here we chose d = 0.9 [59]), and *D* corresponds to a diagonal matrix of n×n where d_ii_ is the sum of weights of interactions between gene i and its neighboring genes in the network. The weights are contained in the matrix W (n×n), where w_ij_ is used to measure the overall similarity between gene i and its neighbor gene j from the aspects of interaction, expression and function. Thus, W was characterized as:

W=α⋅S(i,j)+β⋅P(i,j)+γ⋅F(i,j) (2)

Here S(*i*,*j*),P(*i*,*j*) and F(*i*,*j*) denote the interaction credibility score, Pearson correlation coefficient, and shared functional significance score between gene *i* and gene *j*, respectively. Three coefficients α, β, and γ in the range of [0,1] were used to assess the importance of S(*i*,*j*), P(*i*,*j*), and F(*i*,*j*) in formula, respectively.

The interaction credibility scores S(*i*,*j*) for each pair of gene *i* and *j* was calculated as follows [58]:

$$S(i,j)=C_{ij}^{t}{\left(\frac{C_{ij}C_{••}}{C_{i•}C_{•j}}\right)}^{1-\text{t}}$$ (3)

where C_i•_ and C_•j_ are the sums over all pairs involving *i* or *j* and another entity, C_••_ is the sum over all pairs of entities, C_ij_ represents the sums over all pairs involving both i and j, and t = 0.6 [58]. The parameters were optimized on the KEGG benchmark set [58]. The co-occurrence score C_ij_ was defined as:

$$C_{ij}=\sum_{k=1}^{b}(\delta_{dijk}v_{d}+\delta_{pijk}v_{p}+\delta_{sijk}v_{s})$$ (4)

where v_d_ = 1, v_p_ = 2, and v_s_ = 0.2 are the weights for co-occurrence genes within the same document, paragraph, and sentence based on literature mining, respectively. The delta functions δ_dijk_, δ_pijk,_ and δ_sijk_ are 1 if the genes *i* and *j* are both mentioned in the document k, a paragraph of k or a sentence of k, otherwise they are 0 [58].

The Pearson correlation coefficient P(*i*,*j*), which is used to represent the co-expression relationship between gene *i* and gene *j*, was defined as follows:

$$P(i,j)=\frac{1}{h-1}\sum_{\text{y}=1}^{h}\left(\frac{i_{y}-\bar{i}}{s_{i}})(\frac{j_{y}-\bar{j}}{s_{j}}\right)$$ (5)

where h is the number of normal samples adding recurrent samples in the expression profile, $\bar{i}$, $\bar{j}$, s_i,_ s_j_, i_y_ and j_y_ represent the average expression value of normal and recurrent samples, standard deviation and observed values of i and j, respectively.

For the shared functional significance score F(i,j) between gene i and gene j, one function was represented by one GO term fm. F(i,j) is defined as the total sum of the significance of the functions shared:

$$F(i,j)=\sum_{m=1}^{x}sig(f_{m})$$ (6)

where x is the number of common GO terms annotated by genes i and j, and sig(*f*_m_) denotes the significance of a function fm, which was defined as follows:

$$sig(f_{m})=\frac{1}{\left|Gene(f_{m})\right|}$$ (7)

here Gene(fm) is genes annotated on GO term fm, |Gene(*f*_m_)| is the number of genes annotated to fm. We calculated the ranking score q for each gene in the disease-related network and ranked these genes in the descending order of q.

In the formula (1) and (2), all the combinations of α, β, γ, and d were used to rank candidate genes. The best α, β, γ combination was determined according to the seed genes identified in the top 50 and 100 ranking list. The best d value was selected based on the α, β, and γ combination which showed the best performance in ranking candidate genes.

### Validation

The comparison of FIP with ToppNet/Endeavour was carried out using the same data. The performance of them was assessed using the Leave-One-Out Cross Validation (LOOCV). For all the seed genes, one seed gene was removed as a test gene each time, and then added to candidate genes. All the candidate genes were ranked by our method to determine the ranking of the test gene. This procedure was repeated until all the seed genes were used up as test genes. Receiver Operating Characteristic (ROC) curves were then plotted and the area under ROC curve (AUC) values were used to compare the performances of the three methods.

## Results

### Optimization of ranking coefficient parameters

**Fig 2:**
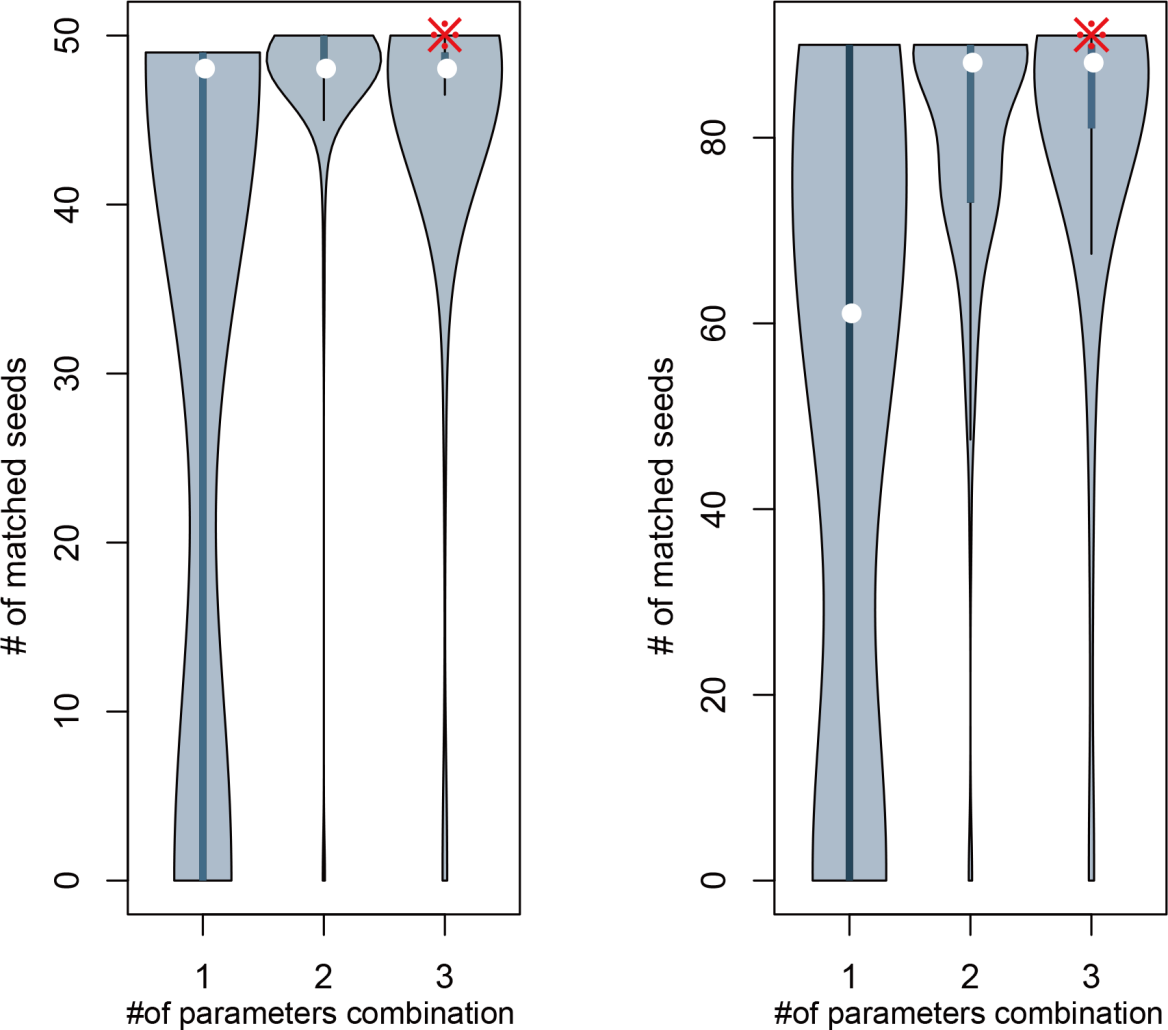
Violin plots of the number of matched seeds identified in top 50 and 100 of the ranking list. The number 1, 2 and 3 represent the number of parameter(s) in the parameter combinations, respectively. The asterisks and white circles present our results and the medians of each combination.

As described in method, score vector Q for all the genes was calculated based on the rankings from the separated data sources such as gene ontology, protein-protein interaction, and gene expression in their corresponding coefficients α, β, and γ, respectively. Candidate genes, which were the common genes in the disease-related network and the differentially expressed genes identified using SAM, were then ranked in the descending order of Q value. For the top 50 and 100 genes in the ranking list, we calculated the number of matched seeds against single, two and three these parameter combination, respectively. There was a significant difference between single and multiple parameter combinations both in top 50 and 100, as well as between two and three parameter combination in top 50 of ranking gene list (t-test, p<0.05) (Fig 2).

The LOOCV has been further applied for all parameter combinations and four parameter combinations (α = 0.8, β = 0.5, γ = 0.9 (AUC = 0. 0.9107); α = 0.7, β = 0.5, γ = 0.8 (AUC = 0.9187); α = 0.9, β = 0.5, γ = 0.8 (AUC = 0.8955); α = 0.9, β = 0.6, γ = 0.8 (AUC = 0.8763)) were shown to be better than the rest. Since no other independent dataset of VTE could be obtained, 10-fold cross-validation was carried out to further select the optimized parameter values in Formulas 1 and 2 from these four parameter combinations (α = 0.8, β = 0.5, γ = 0.9 (AUC = 0. 0.9013); α = 0.7, β = 0.5, γ = 0.8 (AUC = 0.8948); α = 0.9, β = 0.5, γ = 0.8 (AUC = 0.8527); α = 0.9, β = 0.6, γ = 0.8 (AUC = 0.8416)).

**Fig 3:**
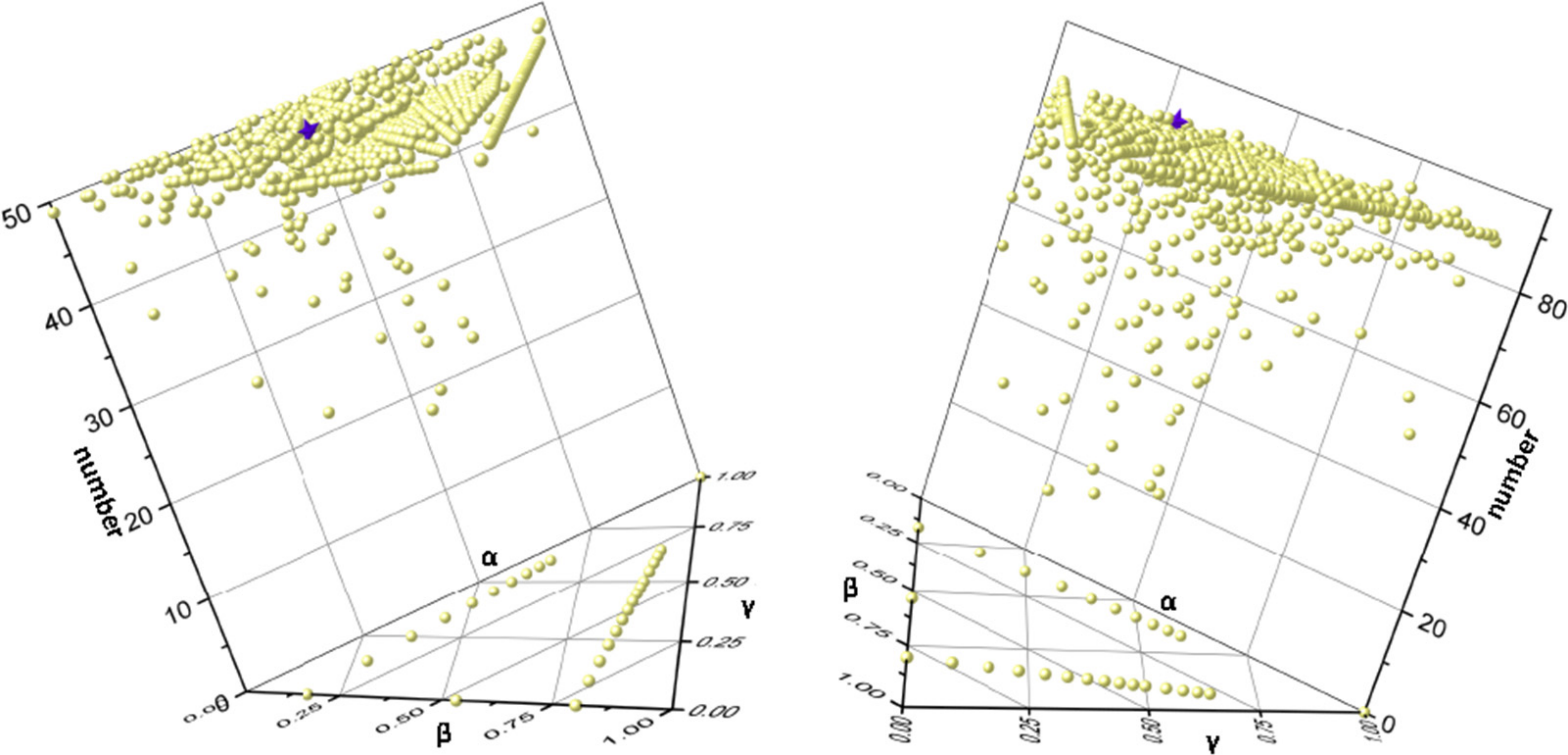
The 3-D distribution of seed genes in top 50 (left) and top 100 (right) of the ranking gene list against all parameter combinations. The three sides of the triangle coordinate system represent the three parameters, respectively. The perpendicular axis of the triangular coordinate system represents the number of seed genes. The purple five-pointed star and yellow ball present the optimal parameter combination and all other parameter combinations.

The optimal parameter combination of α = 0.8, β = 0.5, and γ = 0.9, was achieved (Fig 3).

**Fig 4:**
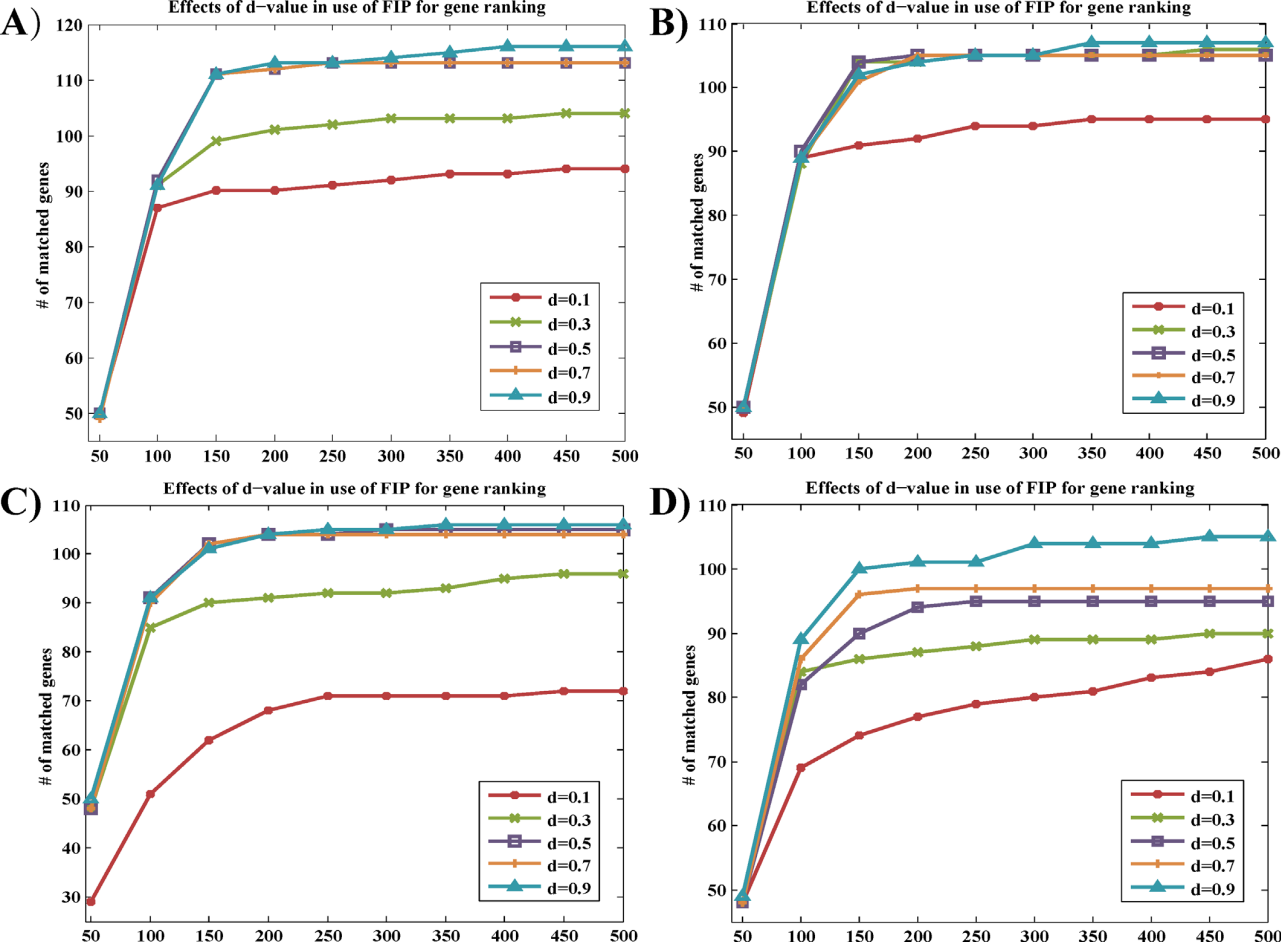
Comparison of performance of FIP-based gene ranking with different d values for four parameter combinations. The number of matched genes identified using FIP with five different d values (d = 0.1, 0.3, 0.5, 0.7, and 0.9) for four parameter combinations were counted and plotted ((A)α = 0.8, β = 0.5, γ = 0.9; (B)α = 0.7, β = 0.5, γ = 0.8; (C)α = 0.9, β = 0.5, γ = 0.8; (D)α = 0.9, β = 0.6, γ = 0.8). Y-axis: the number of matched genes identified using FIP; X-axis: the number of ranked genes.

For all parameter combinations, genes were also ranked according to the calculated q scores with five different d values (d = 0.1, 0.3, 0.5, 0.7, and 0.9). The matching numbers of genes were applied to assess the effectiveness of FIP (Fig 4). The number of matched seeds among top 500 in the ranking list of d = 0.9 was higher than those of other d-values.

Finally, the parameter combination of α = 0.8, β = 0.5, γ = 0.9, and d = 0.9 was selected to calculate vector Q so as to obtain the ranking results.

### Prioritization of candidate genes and validation by literature review

In the disease-related network, all the genes were prioritized by FIP according to vector Q in the optimal ranking coefficient parameter combination. As a result, a total 200 of top candidate genes were generated (S1 Table). We manually searched these top 200 candidate genes for drug targets in literature of PubMed (http://www.ncbi.nlm.nih.gov/pubmed). There were 34 antithrombotic drug targets among the top 200 candidates, including thrombin -activated factor 2 receptor (F2R; rank 5), SELPLG (rank 6), APOA1 (rank 10), SCARB1 (rank 17), TTR (rank 30), and F10 (rank 37) (S1 Table). Thrombin-activated factor 2 receptor (F2R) was reported to link thrombosis to inflammation modulating interleukin 6 (IL6) synthesis [60,61]. Treatment of rats with APOA1 Milano (the mutant form of human APOA1) was shown to markedly delay thrombus formation, inhibit platelet aggregation, and to reduce weight of the thrombus [62]. FX protein was encoded by gene F10, and its mutations gave rise to severe Factor X (FX) deficiency. Anti-FX inhibitor had been approval by FDA for the prevention of venous thromboembolism surgical intervention and as an initial treatment for deep venous thrombosis and pulmonary embolism [63–65].

Non-drug target candidate genes of the top 200 candidates were also reported to be associated with thrombosis. For instance, SNP could be used in the prediction of recurrent thrombosis such as susceptibility gene ALPL (rank 1) with SNP [66,67]. The coagulation factor III gene (F3; rank 11) was suggested to produce tissue factor, which could initiate thrombosis on disrupted atherosclerotic plaques [68]. The loss of CYP2C19 (rank 22) function triggered platelet reactivity, which was a predictor of stent thrombosis [69,70]. Variation of VTN (rank 28) promoter haplotype, causing transcription factor binding activity increased, was proposed to be a novel genetic marker for deep venous thrombosis [71]. Sex hormone-binding globulin (SHBG; rank 51), easily measured in routine laboratories, could serve as a marker for the risk of venous thrombosis [72].

Taken together, of the top 200 candidate genes in the ranking list, 124 candidate genes predicted by our method had been confirmed to be correlated with thrombosis in PubMed literature, which have not been recorded in disease databases (OMIM, GAD and DO) (S1 Table). Top-ranked candidates were found to have a high confirmation rate in terms of their association with thrombosis, especially top 10 candidates (Table 1).

**Table 1:** The confirmation rate of top 200 candidate genes in the ranking list.

Top n	Confirmation Number	Confirmation Rate
10	10	100.00%
20	16	80.00%
40	30	75.00%
50	35	70.00%
100	66	66.00%
150	100	66.67%
200	124	62.00%

The confirmation rate was calculated by dividing the confirmation number by the corresponding number of top n. It represented the effectiveness of the confirmation.

### Validation of FIP through Functional and pathway analysis

**Fig 5:**
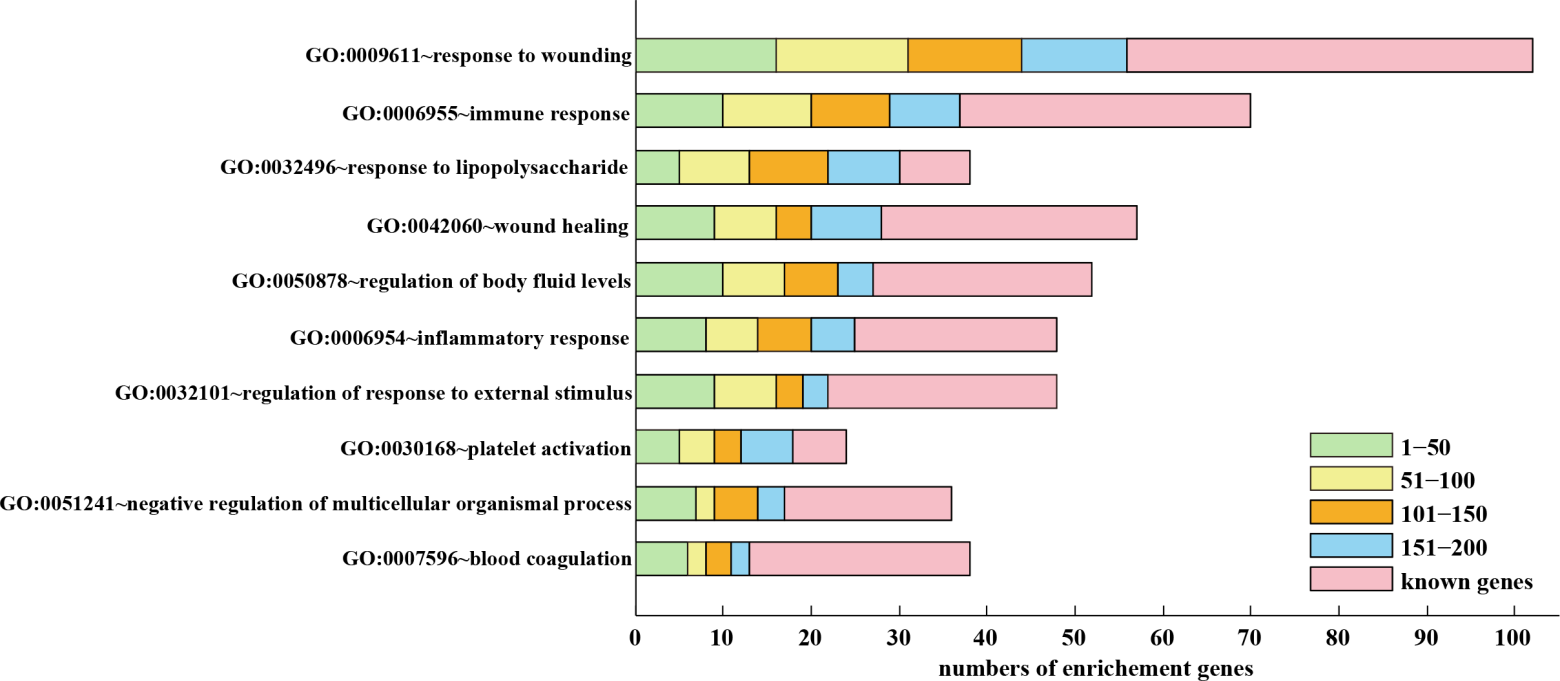
The top 200 candidate genes and known genes involved in the identified 10 functional categories. The genes were analyzed in GO and KEGG with DAVID and classified into 10 VTE-related functional categories.

We conducted DAVID (http://david.abcc.ncifcrf.gov/) [73] and Gene Ontology (GO, http://geneontology.org/) (Biological Process and Molecular Function) [74] analysis to assess the functional enrichment of the identified candidate genes. In this way, the biological features/or meanings of the candidate genes can be extracted in order to improve the classification of these genes in terms of their functionalities. The classification was further interpreted in KEGG (http://www.genome.jp/kegg/) [75] pathways (FDR <0.05). Top 200 candidates were selected and divided into four groups with 50 genes in each, followed by KEGG and GO analysis in DAVID. As a result, 10 significant functional categories were identified and associated with thrombotic disease (Fig 5) [76–83]. For instance, GO: 0007596~blood coagulation was reported to be the main cause of thrombosis and recurrence. Blood coagulation, causing damage to the vascular endothelium, was suggested to initiate acute venous thrombus generation [84]. The maximum number of candidate and seed genes were found in GO: 0009611~respond to wounding functional category. The most common sites of wounding in conflict were extremities, which were associated with a significant incidence of vascular trauma, and had a high complication rate (graft thrombosis) [85]. ‘GO: 0030168~platelet activation’, leading to severe end-organ damage, was shown to increase the risk of thrombosis, implying that platelet reactivity was an important pathological mechanism of thrombosis [86,87].

**Fig 6:**
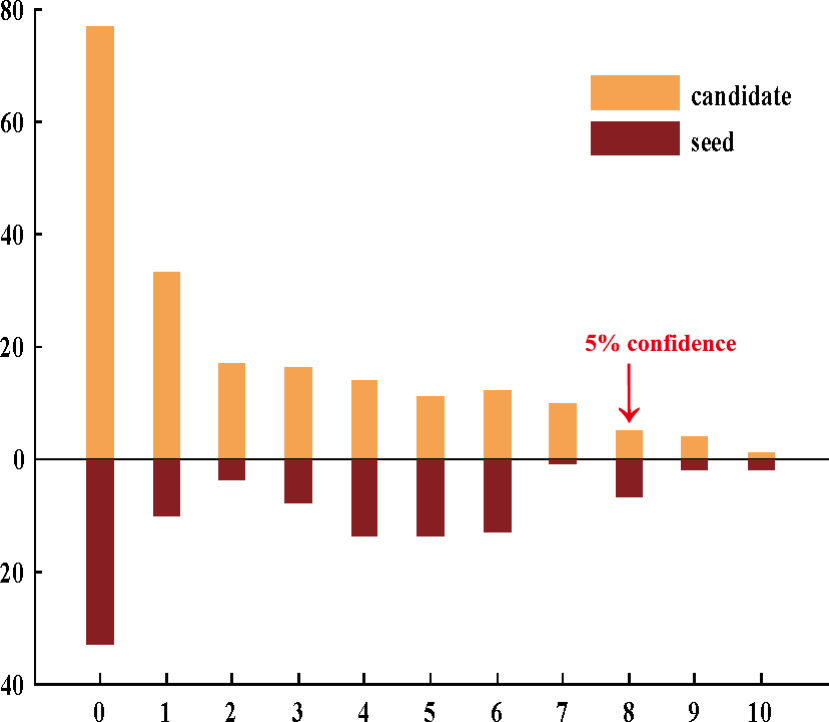
The distribution of the top 200 candidates and seeds among different functional categories. X-axis and y-axis represent the number of the functional categories and enriched genes, respectively.

We counted the number of the candidate and seed genes among the 10 functional categories which each gene was annotated to. Ten candidate genes (95% confidence interval) appeared in more than 8 functional categories and were confirmed by literature (Fig 6).

Moreover, 6 of these candidates were drug targets, and 3 of them were at top 50 candidate genes (Table 2).

**Table 2:** The candidate genes in more than eight functional categories.

Gene	Frequency	Rank	Drug	PubMedID
TLR4	10	105	√	PMID:24488914
APOA1	9	10	√	PMID:18801202
CX3CL1	9	191		PMID:25795074
F2R	9	5	√	PMID:19404549
THBS1	9	57	√	PMID:25343959
A2M	8	41		PMID:20156641
F2RL1	8	31		PMID:12069753
SERPINF2	8	8		PMID:20696792
SYK	8	143		PMID:24376657
VEGFA	8	45	√	PMID:25006132

**Fig 7:**
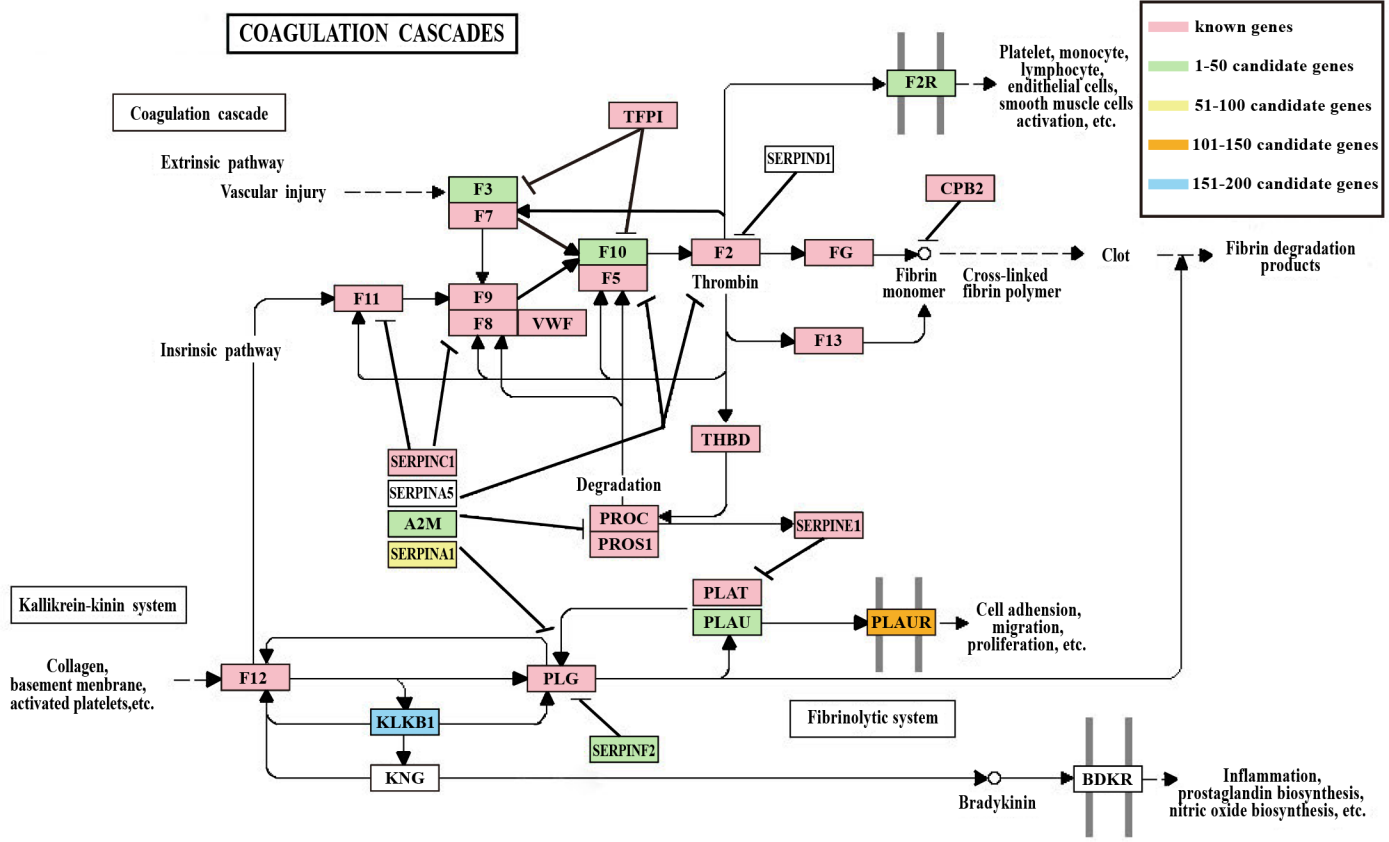
Coagulation cascades pathway. The red, green, pink, orange and blue rectangles present known genes in the top 1–50, top 51–100, top 101–150 and top 151–200 candidate genes.

Furthermore, the known disease-related genes and top 200 candidate genes were obviously enriched in four common pathways: Hematopoietic cell lineage, cytokine-cytokine receptor interaction, Cell adhesion molecules (CAMs) and complement and coagulation cascades pathway (FDR<0.05). The coagulation cascade pathway appeared to be a critical determinant of atherosclerotic plaque thrombogenicity [88]. Cell adhesion molecules (CAMs), hematopoietic cell lineage and cytokine-cytokine receptor interaction were also associated with thrombosis [85,89,90]. We mapped the enriched genes, including the known disease-related genes and candidate genes, in the coagulation cascades pathway [91,92] (Fig 7).

In the map, there are 19 known genes and 9 candidate genes, respectively. Among these 9 candidate genes, each of them was annotated to no less than four functional categories, especially F2R, SERPINF2, and A2M, which were annotated to more than eight functional categories (Fig 6). Triggering tissue factor (F3) and F2R (coagulation pathway sensors) have been shown to influence the vascular microenvironment and angiogenesis respective of clinically apparent thrombosis [93,94]. The mutations of other two genes, PROC and PROS1, were shown to increase risk of recurrent thromboembolic events if they were combined with other genetic or environmental thrombosis factors [95,96]. A2M was reported to inhibit the known genes PROS1 and PROC in the coagulation cascade pathway, which could be associated with recurrent thrombosis.

### Comparison of FIP to ToppNet and Endeavour

**Fig 8:**
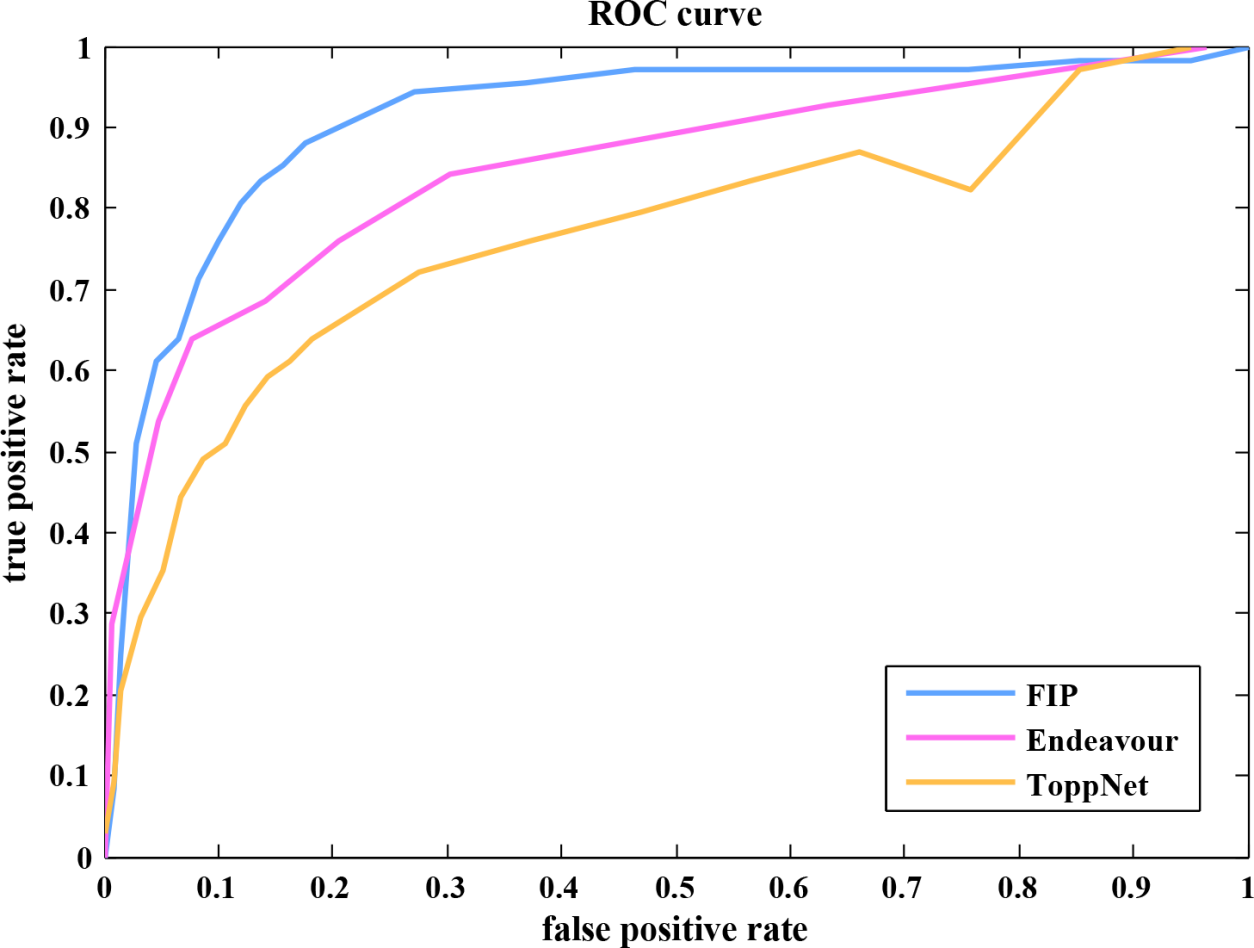
The ROC curves of FIP, ToppNet, and Endeavour methods.

To evaluate the performance of the proposed FIP method in predicting novel recurrent thrombosis genes by prioritizing candidate genes, we carried out LOOCV on the known disease-related genes. In this validation, the same training and testing gene sets were used in the FIP, ToppNet, and Endeavour methods. The ROC curves were plotted to compare the performance of the three methods (Fig 8).

The AUC value of FIP method was 0.9107, which was much higher than ToppNet (0.7150) and Endeavour (0.8127). Thus, FIP method provided a good performance in efficiently identifying known disease-related genes in the prioritization gene list and was more sensitive and specific in ranking the test genes.

**Fig 9:**
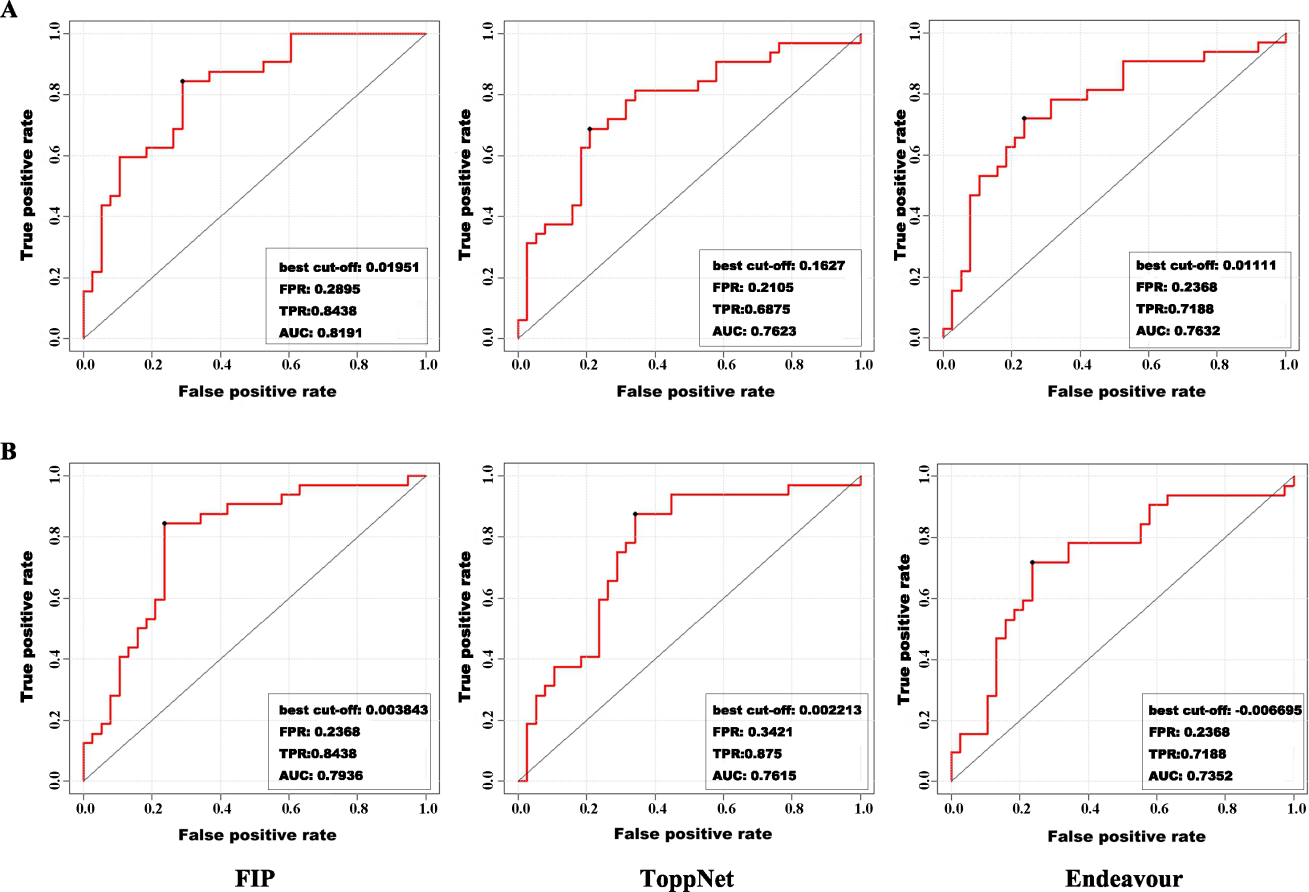
The performance of sample classification with top-ranked candidates of FIP, ToppNet, and Endeavour methods. (A) ROC curves of FIP, ToppNet, and Endeavour methods with the top 50 candidates as classification characteristics (B) ROC curves of FIP, ToppNet, and Endeavour methods with the top 100 candidates as classification characteristics.

To further verify the top-ranked candidates as novel disease recurrence genes, support vector machine (SVM) was applied to classify normal and recurrent samples with top-ranked candidates as classification characteristics. The outcome of FIP was then compared with those of ToppNet and Endeavour methods with the top 50 and top 100 candidates as classification characteristics, respectively. Four performance measurements, false positive rate (FPR), true positive rate (TPR), best cutoff curve, and AUC, were calculated (Fig 9).

The AUC values of FIP were higher than those of ToppNet and Endeavour methods using either the top 50 or 100 candidates as classification characteristics. In the meantime, the AUC values of each method using the top 50 candidates as characteristics were higher than those of each corresponding method using top 100 candidates as characteristics.

**Fig 10:**
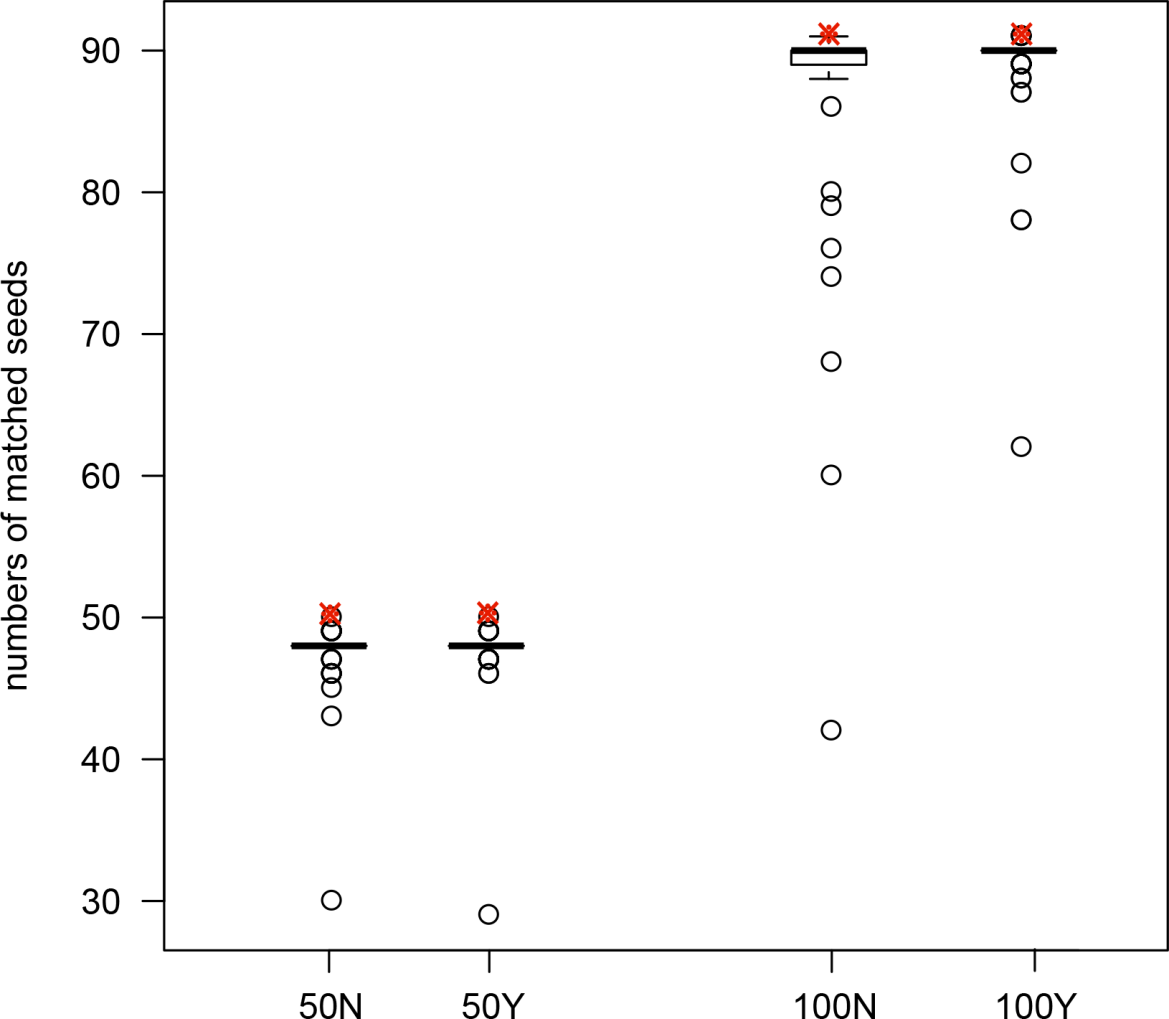
Comparison of the performance of the FIP with and without replacement in random sampling. 50N and 100N were top 50 and top 100 genes in the ranking list with and without replacement, respectively. 50Y and 100Y were top 50 and top 100 genes in the ranking list with back, respectively. Asterisks present our results.

To explore the factors which may affect the performance of FIP, we first assessed the correlation between specific expression profile and outcome of gene prioritization. P(*i*,*j*) in formula (2) were assigned randomly from all correlation coefficients using sampling with and without replacement, respectively. The disease relevance score Q was recalculated and genes were ranked according to the q value. The seed numbers in the top 50 and top 100 ranking list were calculated to evaluate the performance of our method. Each process was repeated 100 times. The results showed that the performance of our method was better than that of random sampling of specific expression data (Fig 10). It suggested that specific expression profile did affect the performance of gene prioritization methods.

**Fig 11:**
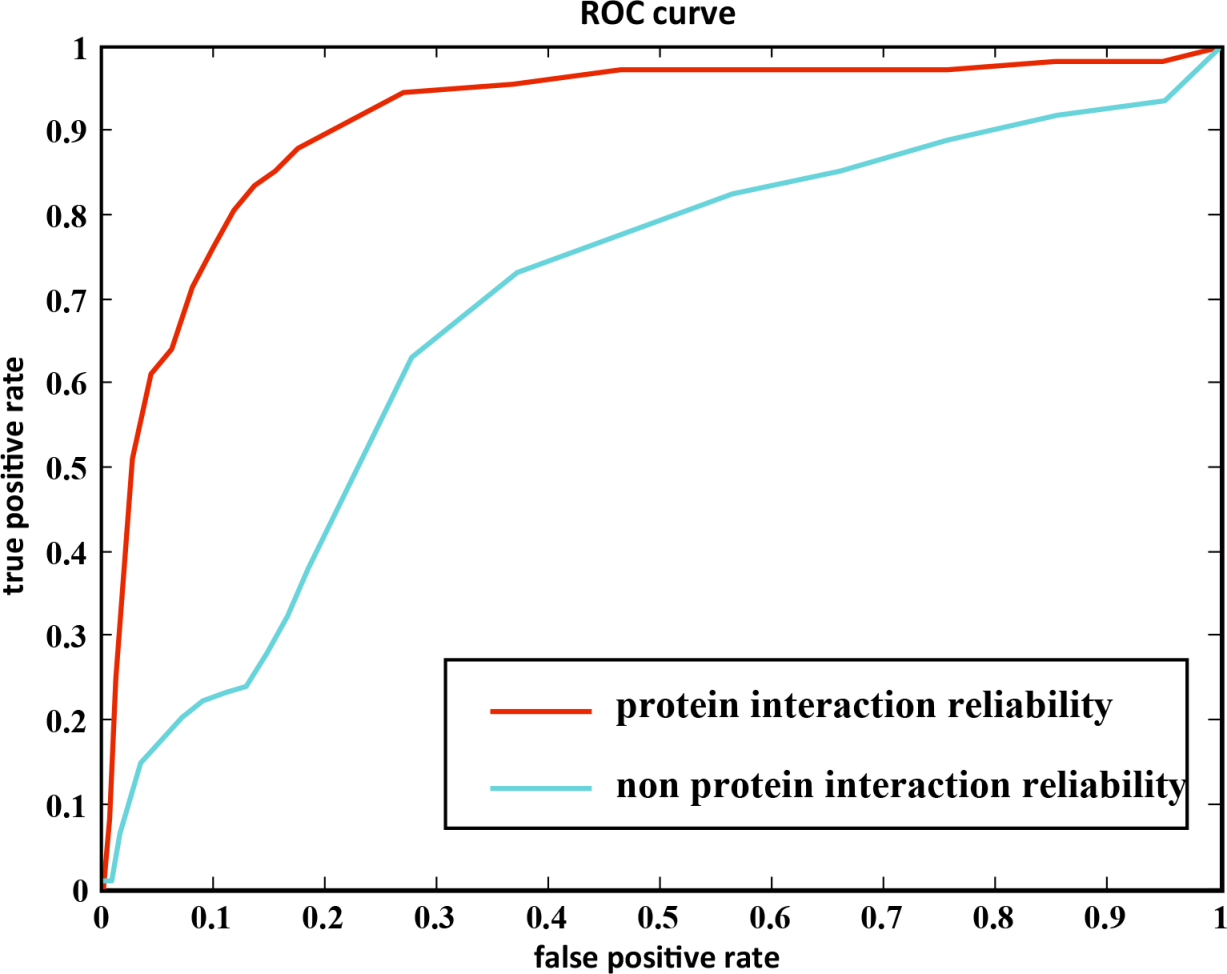
The ROC of the weight with protein interaction reliability and non-protein interaction reliability.

Secondly, we evaluated the importance of protein interaction reliability. We altered the S(*i*,*j*) in formula (2) to 1 (no protein interaction reliability) and recalculated the disease relevance score Q. Genes were ranked according to the q value. LOOCV was used to assess the performance using the new weights. Its AUC (0.6878) was lower than that of the original weights (AUC = 0.9107) (Fig 11).

To evaluate the robustness of FIP, 10-fold CV was also applied to ToppNet and Endeavour. There was a statistical significance between FIP and ToppNet (one-side t-test, p-value<0.05) as well as FIP and Endeavour (one-side t-test, p-value<0.05) (S1 Fig).

We performed literature validation, function annotation and pathway analysis for top 200 candidates of ToppNet and Endeavour (S2–S4 Figs). In general, the performances of FIP were better than those of ToppNet and Endeavour.

## Discussion

In this study, we devised and implemented a novel algorithm called FIP to prioritize candidate genes involved in VTE. This algorism is based on overall similarity with its neighboring genes by taking into account three aspects: expressions, functions, and interactions. In this way, we were able to prioritize the genes involved in VTE. For the top 200 candidates, we manually searched in PubMed literature and 124 genes were confirmed, in which 34 were drug targets. Furthermore, we conducted KEGG and GO analysis to functionally enrich the identified candidate genes. More candidates not confirmed by literature were classified into 10 significant functional categories which were associated with thrombotic disease (Fig 5). Overall FIP had a better predictive performance and achieved a reliable AUC value.

In reality, multiple properties of genes could be associated with each other in disease states contributing to the formation of disease. Integrating multiple data sources of genes has been reported to be better than a single data source in terms of sensitivity and accuracy of gene prioritization [97]. In our study, we compared the performance of integrating three data sources with those of integrating the two. As a result, there was no significant difference of the number of the matched seed genes between the combinations of integrating two data sources, while the combinations of integrating three data sources produced the much better performance than those of integrating two data sources (t-test, p<0.05) in terms of the number of the matched seed genes (S2 Table). Moreover, coefficients such as α, β, and γ and the control parameter d were shown to affect the performance of gene prioritization. According to the number of matched seed genes, LOOCV and 10-fold cross-validation, the best performance of gene prioritization was achieved in the parameter combination of α = 0.8, β = 0.5, γ = 0.9, and d = 0.9 in prioritizing VTE-related genes in this study.

ToppNet and Endeavour are currently commonly used prioritization methods. According to network properties-based knowledge, ToppNet employs three algorithms (PageRank, Hyperlink-Induced Topic Search-HITS, and K-step Markov) to prioritize disease-related candidate genes by estimating their relative importance in PPIN [98,99]. Thus, ToppNet ranks or prioritizes genes based on topological features in PPIN with only one data type. As described above, the performance of integrating more data sources was better than those of integrating less ones. Thus, it is not surprised that FIP outperformed ToppNet in prioritizing genes involved in VTE in this study (Fig 8).

Endeavour takes the similar three data types as what we used in this study to rank candidate genes except its expression data background (high-density gene expression database). As compared to Endeavour, FIP applied disease-specific expression data, including recurrent VTE sample data, in our study. In theory, whether disease-specific or non disease-specific expression data through random sampling gene expression data could affect the performance of gene prioritization. This was confirmed by our results that disease-specific gene expression data did affect the performance of FIP (Fig 10). It was shown that FIP using VTE-specific gene expression data achieved the better performance than Endeavour using non disease-specific expression data since the identified top candidates by FIP through VTE-specific gene express analysis were more likely to be associated with VTE.

On the other hand, protein interaction databases used by the commonly used prioritization methods, including Endeavour, don’t provide the details enough to assess whether a protein binds its interaction partner(s) which share similar structural or chemical properties and functionalities since many protein-protein links are loose because of random or unspecific bindings of proteins collected in databases. Thus, the reliability of protein-protein interaction is interrogated, resulting in low accuracy of ranking genes. In fact, the edge weight in the disease-related network can provide reasonable and consistent values to quantify the strength of connection of proteins. In our study, we took this feature into account in prioritizing candidates. As a baseline for weighted networks, we constructed a non-weighted network with the same protein interaction pairs and assessed the performance of FIPs using a weighted or a non-weighted network. It was showed FIP using a weighted network achieved a better performance (Fig 11). This result implied that the improved reliability of protein interaction applied by FIP might enhance its performance compared to Endeavour in prioritizing candidates related to VTE.

In summary, our FIP method combined experimental data with mathematical modeling and provided an alternative system biology approach in promising to tackle complex VTE disease for aiding diagnosis of recurrent VTE. This method could also be applied to other complex diseases to reveal disease mechanism and provide new perspective for diagnosis and drug development.

## Supporting Information

S1 Fig The boxplot of FIP, ToppNet and Endeavour. (TIF)

S2 Fig The Venn diagrams of literature validation among three methods at top 100 (left) and top 200 (right) candidates. The numbers in the slash left and right present the number of confirmed genes and the number of candidate genes, respectively.
(TIF)

S3 Fig The comparison of literature validation for top 200 candidates generated from three methods on ten function categories. (TIF)

S4 Fig The comparison of literature validation among three methods at four pathways for top 200 candidates. (TIF)

S5 Fig The Venn diagrams of literature validation among four parameter combinations for top 200 candidates. The numbers in the slash left and right present the number of confirmed genes and the number of candidate genes, respectively.
(TIF)

S1 Table Top 200 candidate genes identified by FIP. (DOC)

S2 Table The significance between combinations among the integration of the two and three data sources. (DOC)

S3 Table The literature validation of top 200 candidate genes among three methods. (DOC)

S4 Table The top 200 candidates of three methods on ten function categories. (DOC)

S5 Table The top 200 candidates among three methods at four pathways. (DOC)

S6 Table The literature validation among four parameter combinations for top 200 candidates. (DOC)
