# Supplementary figures and images for: A Novel Prioritization Method in Identifying Recurrent Venous Thromboembolism-Related Genes
Source: PLoS One. 2016 Apr 6;11(4):e0153006. doi: 10.1371/journal.pone.0153006 (PMC4822849; doi:10.1371/journal.pone.0153006)

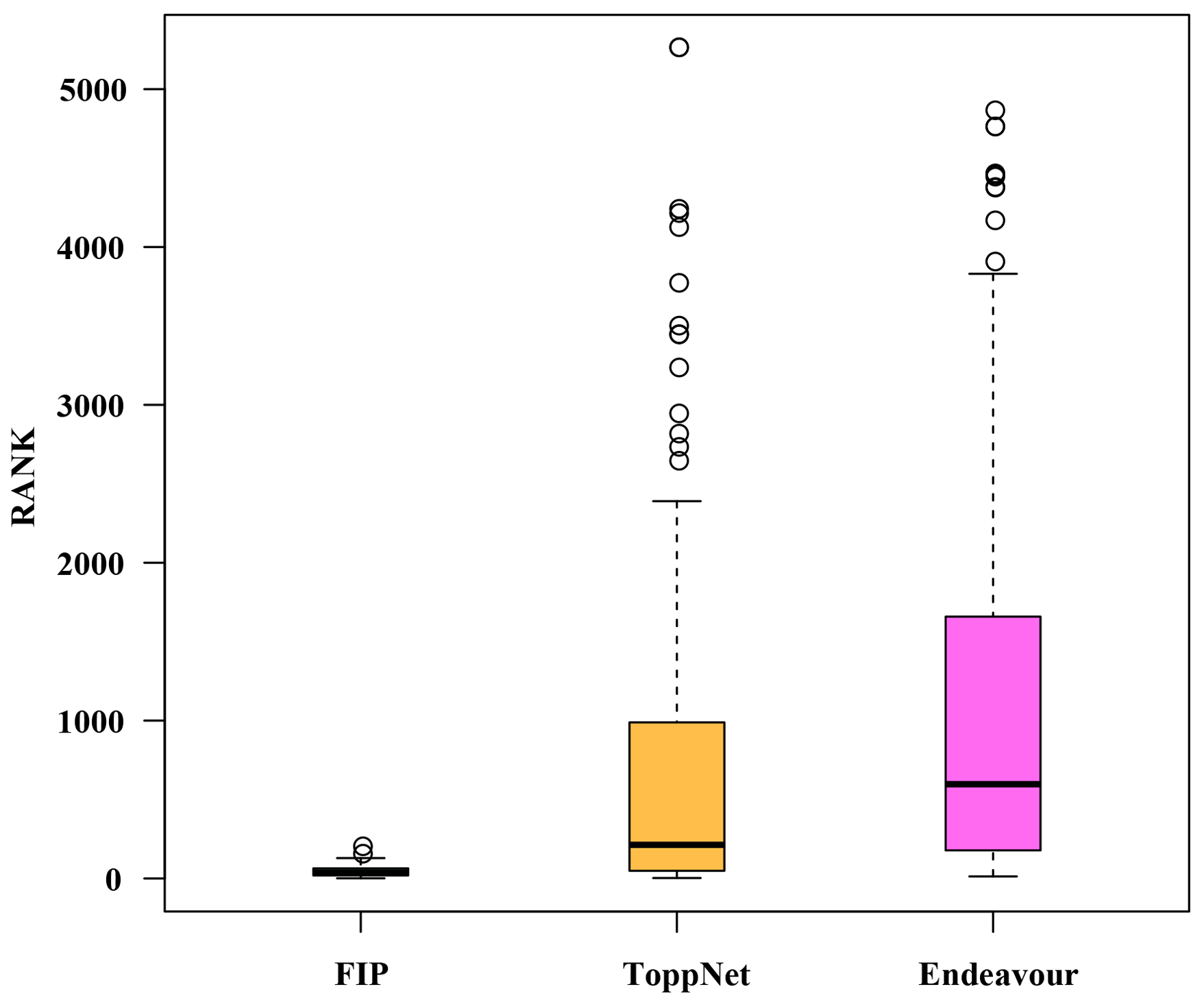

Supplement: S1 Fig — (TIF) [file pone.0153006.s001.tif]

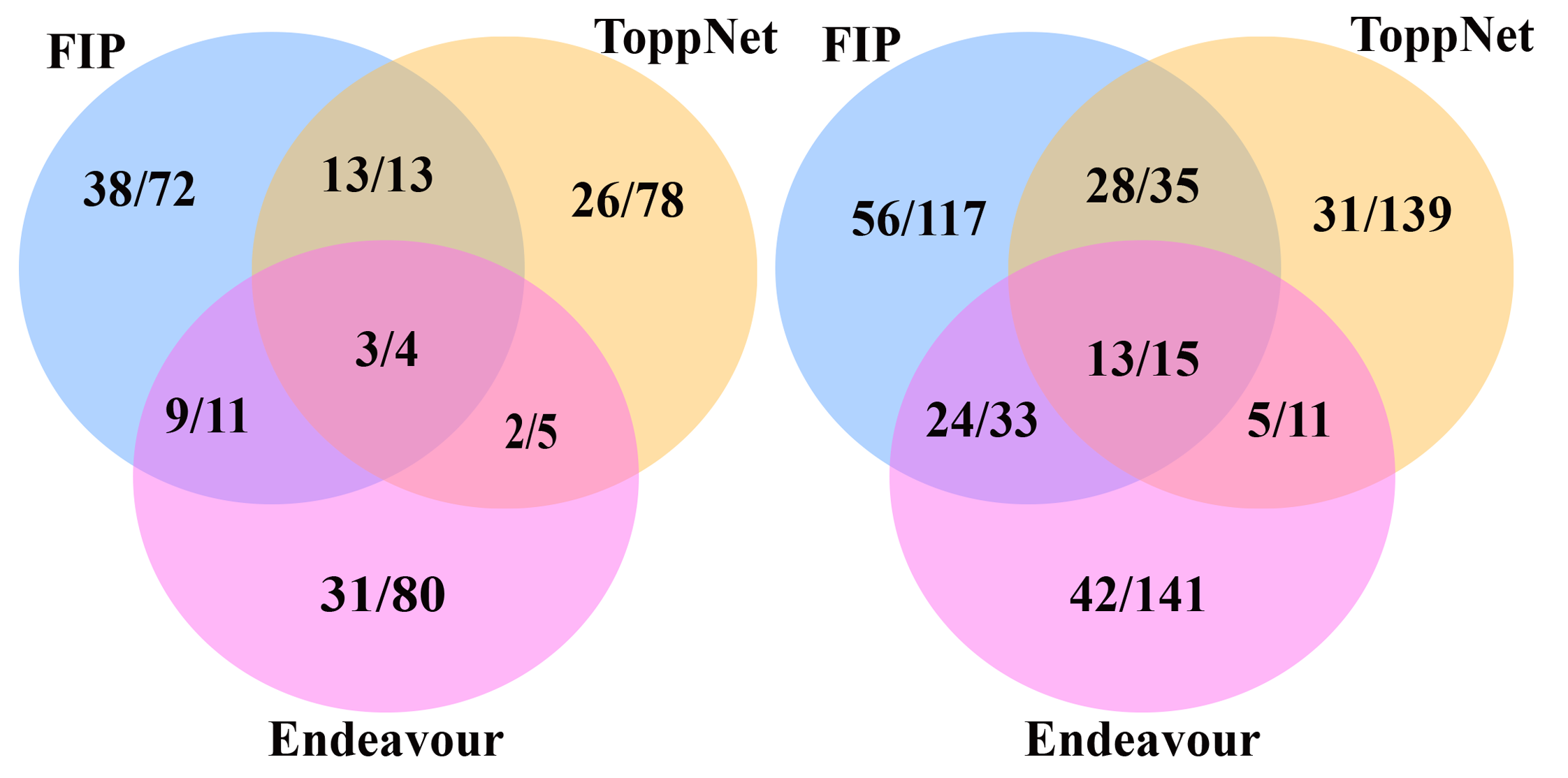

Supplement: S2 Fig — The numbers in the slash left and right present the number of confirmed genes and the number of candidate genes, respectively. (TIF) [file pone.0153006.s002.tif]

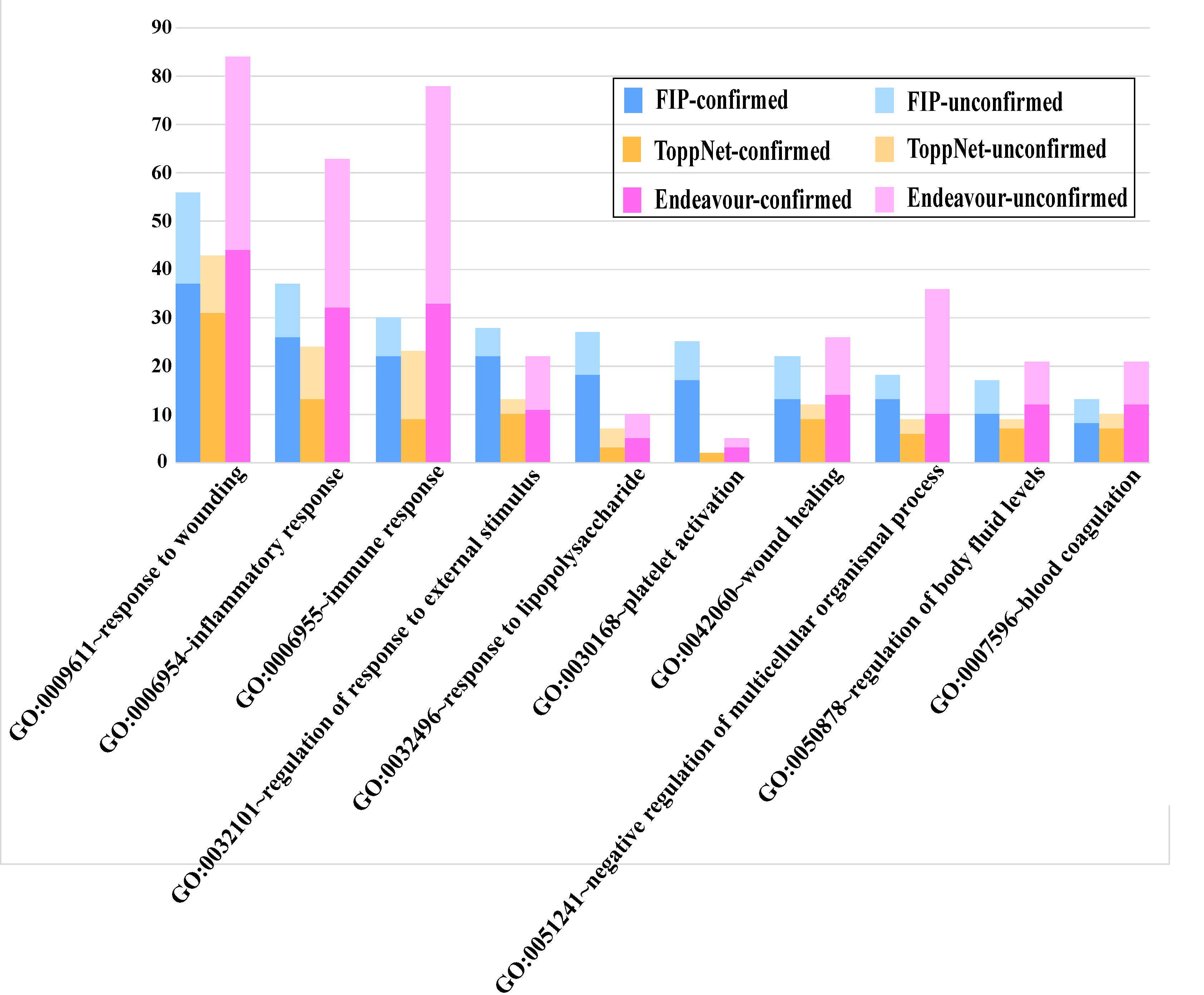

Supplement: S3 Fig — (TIF) [file pone.0153006.s003.tif]

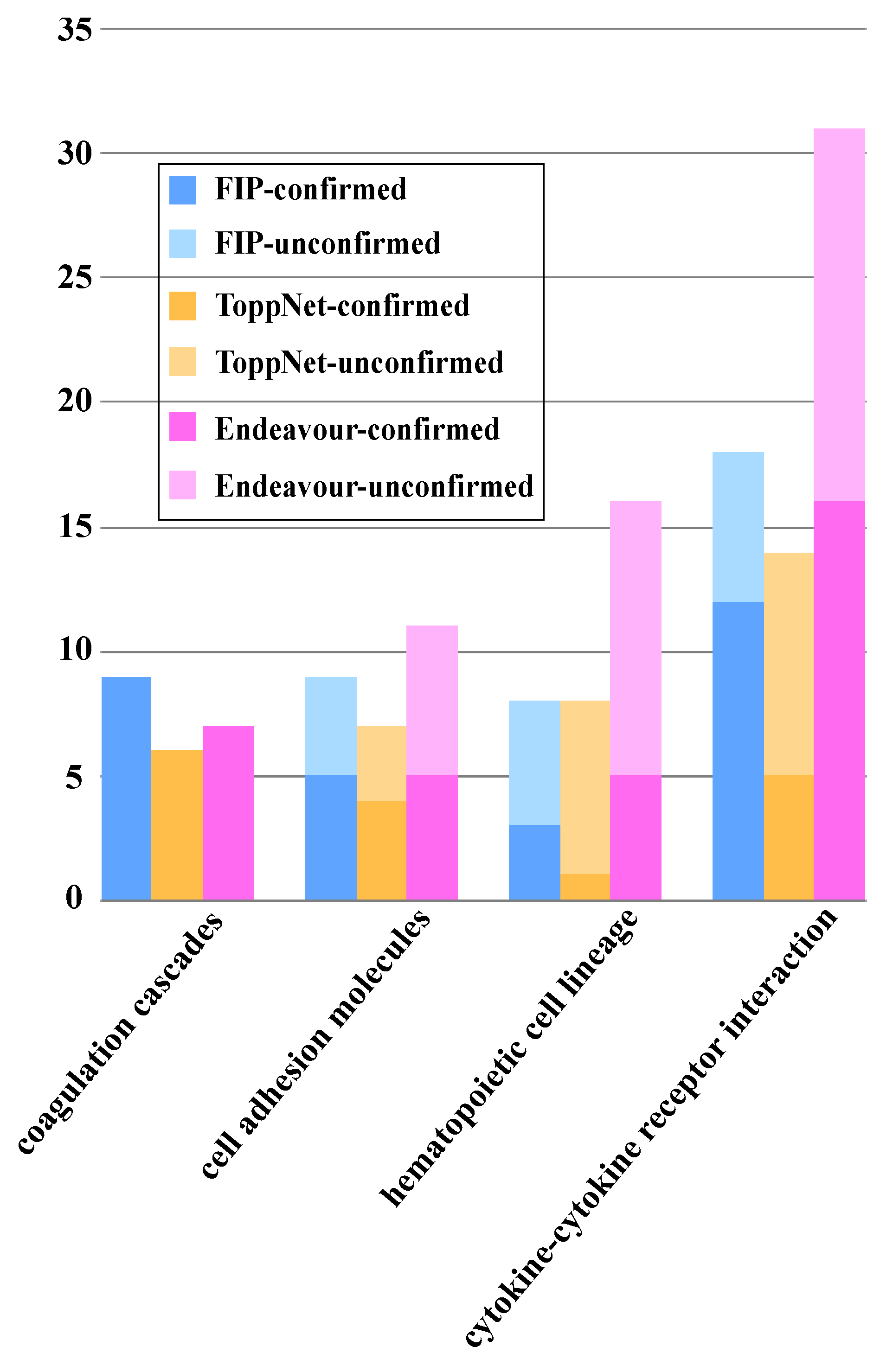

Supplement: S4 Fig — (TIF) [file pone.0153006.s004.tif]

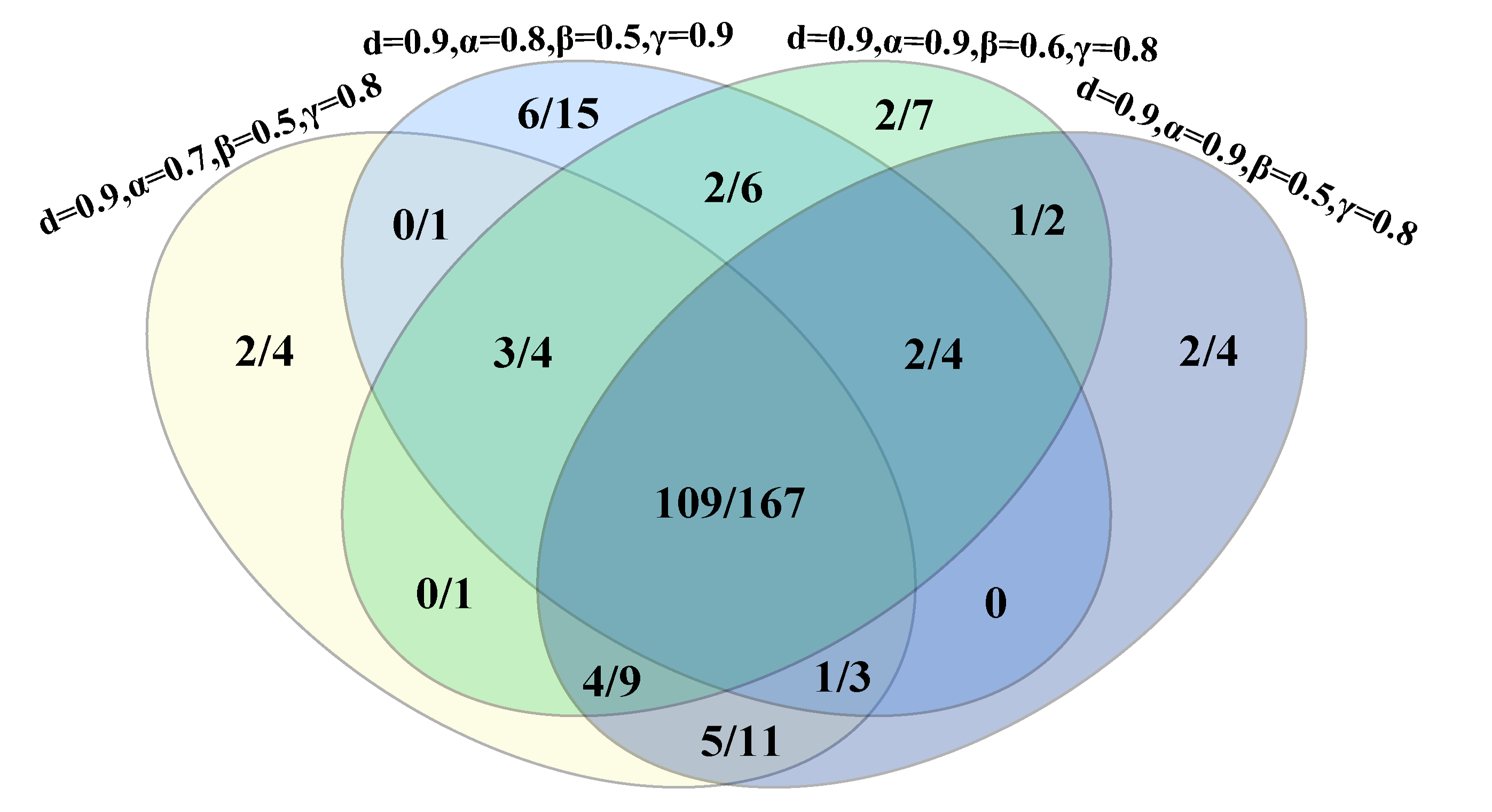

Supplement: S5 Fig — The numbers in the slash left and right present the number of confirmed genes and the number of candidate genes, respectively. (TIF) [file pone.0153006.s005.tif]
