# Supplementary material for: A Novel Prioritization Method in Identifying Recurrent Venous Thromboembolism-Related Genes
Source: PLoS One. 2016 Apr 6;11(4):e0153006. doi: 10.1371/journal.pone.0153006 (PMC4822849; doi:10.1371/journal.pone.0153006)
Supplement: S1 Table — (DOC) [file pone.0153006.s006.doc]

**S1 Table. Top 200 candidate genes identified by FIP .**

| **Rank** | **Gene** | **Score** | **Literature** | **Drug target** |
| --- | --- | --- | --- | --- |
| 1 | ALPL | 816.616 | PMID: 22672431 |  |
| 2 | FGF2 | 658.615 | PMID: 23012273 |  |
| 3 | TCF7L2 | 602.105 | PMID: 24829485 |  |
| 4 | ITGA2B | 593.753 | PMID: 26773046 |  |
| 5 | F2R | 526.302 | PMID: 26446588 | √ |
| 6 | SELPLG | 462.520 | PMID: 18182036 | √ |
| 7 | C5AR1 | 460.960 | PMID: 25830298 |  |
| 8 | SERPINF2 | 456.799 | PMID: 20696792 |  |
| 9 | C3AR1 | 440.966 | PMID: 21526204 |  |
| 10 | APOA1 | 362.588 | PMID: 22955992 | √ |
| 11 | F3 | 342.683 | PMID: 26383585 |  |
| 12 | ACADSB | 329.758 |  |  |
| 13 | CD4 | 302.219 | PMID: 25001165 |  |
| 14 | KIF23 | 288.056 |  |  |
| 15 | SRC | 287.199 | PMID: 26320263 |  |
| 16 | VCAM1 | 274.841 | PMID: 18402813 |  |
| 17 | SCARB1 | 274.645 | PMID:22652597 | √ |
| 18 | FN1 | 271.834 |  |  |
| 19 | PLAU | 261.086 | PMID: 25790727 |  |
| 20 | REN | 258.022 |  |  |
| 21 | POLD1 | 249.609 |  |  |
| 22 | CYP2C19 | 246.776 | PMID: 26147597 |  |
| 23 | CYP2B6 | 246.089 | PMID: 24245489 |  |
| 24 | CYP3A43 | 243.152 |  |  |
| 25 | AKT1 | 241.905 | PMID: 21821713 |  |
| 26 | EGFR | 239.730 | PMID: 26236616 |  |
| 27 | POLE | 239.526 |  |  |
| 28 | VTN | 237.314 | PMID: 23041018 |  |
| 29 | KIF20A | 235.804 |  |  |
| 30 | TTR | 229.671 |  | √ |
| 31 | F2RL1 | 226.324 | PMID:12069753 |  |
| 32 | PRL | 225.390 | PMID: 7697624 |  |
| 33 | APOA2 | 221.608 | PMID: 24829485 |  |
| 34 | MMP9 | 217.640 | PMID: 23490298 | √ |
| 35 | CYP2J2 | 216.642 | PMID:[17533030](http://www.ncbi.nlm.nih.gov/pubmed/17533030) | √ |
| 36 | MMP2 | 215.682 | PMID: 23490298 | √ |
| 37 | F10 | 212.837 | PMID: 22008904 | √ |
| 38 | APOH | 212.451 | PMID: 25081279 |  |
| 39 | STAT3 | 212.008 | PMID: 24736319 |  |
| 40 | ITGAM | 205.963 |  |  |
| 41 | A2M | 205.492 | PMID: 20156641 |  |
| 42 | JUN | 204.907 |  |  |
| 43 | TICAM1 | 203.167 |  |  |
| 44 | ELN | 200.723 |  |  |
| 45 | VEGFA | 199.300 | PMID: 25006132 | √ |
| 46 | HPX | 196.853 | PMID: 24373343 |  |
| 47 | EDN1 | 196.071 | PMID: 23476046 | √ |
| 48 | MPO | 191.847 | PMID: 23818485 | √ |
| 49 | CSF2RB | 191.676 |  |  |
| 50 | CSN1S1 | 189.572 |  |  |
| 51 | SHBG | 188.767 | PMID: 26700933 |  |
| 52 | EDC3 | 188.121 |  |  |
| 53 | IL10RA | 184.418 | PMID: 19134193 |  |
| 54 | PPARA | 181.069 | PMID: 21226266 | √ |
| 55 | POLA1 | 180.575 |  |  |
| 56 | POMC | 178.691 |  |  |
| 57 | THBS1 | 170.872 | PMID: 25343959 | √ |
| 58 | SST | 169.937 | PMID: 25644017 |  |
| 59 | MAPK3 | 169.527 | PMID: 17332680 |  |
| 60 | KLF2 | 162.907 | PMID: 25039491 |  |
| 61 | GOT2 | 162.030 |  |  |
| 62 | MMP14 | 161.390 | PMID: 16171603 |  |
| 63 | MAPK1 | 160.572 | PMID: 18471985 |  |
| 64 | CFTR | 160.110 | PMID: 23506284 |  |
| 65 | IGF2BP3 | 159.451 |  |  |
| 66 | PDE4A | 159.279 |  |  |
| 67 | PLA2G2A | 158.874 |  |  |
| 68 | RELA | 158.736 |  |  |
| 69 | GSTA1 | 158.328 |  |  |
| 70 | SELL | 157.968 |  |  |
| 71 | ERBB2 | 157.281 | PMID: 26221378 |  |
| 72 | IL9R | 154.640 |  |  |
| 73 | LCAT | 154.580 | PMID: 6399344 | √ |
| 74 | DCN | 154.272 | PMID: 24947404 |  |
| 75 | HBEGF | 152.937 | PMID: 22402363 |  |
| 76 | CD79A | 151.660 | PMID: 17317545 |  |
| 77 | TNFRSF1B | 149.113 |  |  |
| 78 | GPX5 | 148.768 |  |  |
| 79 | APCS | 148.652 |  |  |
| 80 | RAC3 | 148.044 |  |  |
| 81 | SOCS3 | 147.280 | PMID: 19132239 |  |
| 82 | NPY | 144.999 | PMID: 18054939 | √ |
| 83 | IGFBP1 | 144.720 |  |  |
| 84 | CD40LG | 143.695 | PMID: 25908768 |  |
| 85 | FTH1 | 143.305 |  |  |
| 86 | APOC1 | 143.150 | PMID: 23579966 | √ |
| 87 | SERPINA1 | 142.787 | PMID: 23188791 |  |
| 88 | APP | 142.324 | PMID: 17172227 |  |
| 89 | PTGS1 | 142.158 | PMID: 26559689 | √ |
| 90 | ITGB2 | 141.914 | PMID: 26188538 |  |
| 91 | KDR | 141.507 | PMID: 22626841 |  |
| 92 | GPX1 | 140.766 | PMID: 23426106 | √ |
| 93 | CALR | 139.769 | PMID: 25761617 |  |
| 94 | FURIN | 139.624 | PMID: 16493485 |  |
| 95 | UBC | 139.571 |  |  |
| 96 | NFKB1 | 138.951 | PMID: 19095643 |  |
| 97 | CFI | 138.837 | PMID: 23555663 |  |
| 98 | MBL2 | 137.329 | PMID: 25482922 |  |
| 99 | CYP2E1 | 137.281 |  |  |
| 100 | EEF1A2 | 136.680 | PMID: 11412682 |  |
| 101 | TNFRSF1A | 136.668957 | PMID: 20671416 | √ |
| 102 | G6PD | 135.999557 | PMID: 24134175 |  |
| 103 | APOBEC3G | 135.556315 |  |  |
| 104 | ALOX5 | 135.006701 | PMID: 24366255 | √ |
| 105 | TLR4 | 134.469813 | PMID: 26571395 | √ |
| 106 | AVP | 134.132659 |  |  |
| 107 | MAPK8 | 133.209535 |  |  |
| 108 | AR | 132.205587 | PMID: 22005299 | √ |
| 109 | STAT6 | 131.6249 | PMID: 25767272 |  |
| 110 | FOS | 131.09578 | PMID: 15201277 |  |
| 111 | CYP2D6 | 130.689129 | PMID: 23579966 |  |
| 112 | PLAUR | 129.652743 | PMID: 19552680 |  |
| 113 | RNASE1 | 129.606715 | PMID: 23328880 |  |
| 114 | OSM | 129.391543 | PMID: 11460521 |  |
| 115 | PTGIR | 128.419567 | PMID: 24557578 |  |
| 116 | TIMP2 | 128.292478 | PMID: 22344262 |  |
| 117 | F2RL2 | 128.132044 | PMID: 24349080 |  |
| 118 | TP53 | 127.975372 | PMID: 26565403 |  |
| 119 | TGFBI | 127.10568 |  |  |
| 120 | MMP12 | 126.965095 | PMID: 15845912 |  |
| 121 | AFP | 126.554658 | PMID: 26576554 |  |
| 122 | HLA-C | 126.402466 | PMID: 1810703 |  |
| 123 | TG | 126.050036 | PMID: 22955992 |  |
| 124 | CRH | 124.357662 |  |  |
| 125 | AGER | 124.20479 | PMID: 24203067 | √ |
| 126 | SERPINA6 | 124.119576 |  |  |
| 127 | ACTA2 | 122.528569 | PMID: 22946110 |  |
| 128 | MMP1 | 122.356399 | PMID: 23814055 | √ |
| 129 | FGFR2 | 121.537941 |  |  |
| 130 | LPAR2 | 120.874615 |  |  |
| 131 | CYP11B2 | 119.883304 | PMID: 21228735 | √ |
| 132 | RAC1 | 118.771771 | PMID: 25628054 |  |
| 133 | CASR | 118.764479 |  |  |
| 134 | F2RL3 | 118.57929 |  |  |
| 135 | CLU | 118.313725 | PMID: 24758255 | √ |
| 136 | IL13RA1 | 117.152731 |  |  |
| 137 | ABCG5 | 117.138477 | PMID: 24166850 |  |
| 138 | MAS1 | 116.608091 |  |  |
| 139 | HLA-B | 116.554889 | PMID: 21930318 | √ |
| 140 | UCP2 | 116.027062 | PMID: 18839467 | √ |
| 141 | AGXT | 115.848384 |  |  |
| 142 | TLR2 | 115.449126 | PMID: 26476743 | √ |
| 143 | SYK | 115.422973 | PMID: 26518435 |  |
| 144 | GFAP | 114.654982 | PMID: 25043249 |  |
| 145 | CTSB | 114.605811 |  |  |
| 146 | JAK3 | 114.583821 | PMID: 15180539 |  |
| 147 | GSTA4 | 113.403526 |  |  |
| 148 | HNF1A | 113.150008 | PMID: 12911579 |  |
| 149 | FASN | 111.535283 | PMID: 22675511 |  |
| 150 | MTTP | 111.442782 |  | √ |
| 151 | LIPE | 111.289536 |  |  |
| 152 | CTSD | 110.964147 | PMID: 19910633 |  |
| 153 | MGP | 110.943543 | PMID: 26040031 |  |
| 154 | CCK | 110.79294 | PMID: 11330425 |  |
| 155 | IFNG | 110.673426 | PMID: 26690514 |  |
| 156 | SULT2B1 | 109.818355 |  |  |
| 157 | LDLRAP1 | 109.788177 |  |  |
| 158 | CDH5 | 109.710851 |  |  |
| 159 | OTC | 109.40194 | PMID: 19343772 |  |
| 160 | TH | 108.357803 |  |  |
| 161 | CTGF | 108.127246 | PMID: 25462173 |  |
| 162 | ENG | 108.11914 |  |  |
| 163 | ABCB11 | 107.734725 |  |  |
| 164 | MASP2 | 107.087246 | PMID: 25533914 |  |
| 165 | SLC10A1 | 107.01959 |  |  |
| 166 | MBP | 106.891235 | PMID: 20888633 |  |
| 167 | HPR | 106.163567 |  |  |
| 168 | CDH2 | 105.942227 |  |  |
| 169 | CYP4A11 | 105.838338 | PMID: 17405690 |  |
| 170 | CD80 | 105.630041 | PMID: 12682232 |  |
| 171 | EPHX1 | 105.320374 |  |  |
| 172 | GP1BA | 104.999356 | PMID: 22872156 | √ |
| 173 | ITIH4 | 104.563551 |  |  |
| 174 | MYD88 | 104.119085 | PMID: 25738377 |  |
| 175 | CMA1 | 104.09825 |  | √ |
| 176 | BHMT | 103.953252 | PMID: 22192524 |  |
| 177 | CCL17 | 103.898424 | PMID: 18723831 |  |
| 178 | ADRBK1 | 103.164006 |  |  |
| 179 | GPX4 | 102.965613 | PMID: 23770613 |  |
| 180 | DRD2 | 102.678774 |  |  |
| 181 | FETUB | 102.560078 |  |  |
| 182 | MAPK14 | 102.038858 |  |  |
| 183 | IL5 | 101.94502 | PMID: 24178511 |  |
| 184 | GAST | 101.836518 |  |  |
| 185 | GH1 | 101.352697 | PMID: 21273694 |  |
| 186 | TNNT2 | 100.902297 |  |  |
| 187 | BGN | 100.811692 |  |  |
| 188 | CD19 | 100.696642 | PMID: 21571767 |  |
| 189 | KLKB1 | 100.08479 | PMID: 25684211 |  |
| 190 | VEGFC | 99.339789 | PMID: 16621967 |  |
| 191 | CX3CL1 | 98.872997 | PMID: 22244861 |  |
| 192 | CLEC3B | 98.190244 |  |  |
| 193 | RXRA | 97.97804 |  |  |
| 194 | RNASE2 | 97.416078 |  |  |
| 195 | TNC | 97.216627 |  |  |
| 196 | UCP1 | 97.181014 | PMID: 16844662 |  |
| 197 | IL6R | 96.39909 | PMID: 22421340 |  |
| 198 | PIK3CD | 96.367988 |  |  |
| 199 | ITIH2 | 96.34373 |  |  |
| 200 | PTPN1 | 96.218806 | PMID: 25042561 | √ |
