# Supplementary material for: A Novel Prioritization Method in Identifying Recurrent Venous Thromboembolism-Related Genes
Source: PLoS One. 2016 Apr 6;11(4):e0153006. doi: 10.1371/journal.pone.0153006 (PMC4822849; doi:10.1371/journal.pone.0153006)
Supplement: S2 Table — (DOC) [file pone.0153006.s007.doc]

**S2 Table. The significance between combinations among the integration of the two and three data sources.**

| **Combination** | **All and PI** | **All and IF** | **All and PF** | **PI and IF** | **PI and PF** | **IF and PF** |
| --- | --- | --- | --- | --- | --- | --- |
| **P(one-tailed)** | 0.016 | 0.002 | 0.038 | 0.5 | 0.053 | 0.063 |
| **P(two-tailed)** | 0.033 | 0.004 | 0.075 | 1 | 0.105 | 0.126 |

All: The combination among the integration of the three data sources.

PI: The combination among the integration of the expression and interaction data sources.

IF: The combination among the integration of the interaction and function data sources.

PF: The combination among the integration of the expression and function data sources.
