# Supplementary material for: A Novel Prioritization Method in Identifying Recurrent Venous Thromboembolism-Related Genes
Source: PLoS One. 2016 Apr 6;11(4):e0153006. doi: 10.1371/journal.pone.0153006 (PMC4822849; doi:10.1371/journal.pone.0153006)
Supplement: S3 Table — (DOC) [file pone.0153006.s008.doc]

**S3 Table. The literature validation of top 200 candidate genes among three methods.****Error: Reference source not found**
