# Supplementary material for: A Novel Prioritization Method in Identifying Recurrent Venous Thromboembolism-Related Genes
Source: PLoS One. 2016 Apr 6;11(4):e0153006. doi: 10.1371/journal.pone.0153006 (PMC4822849; doi:10.1371/journal.pone.0153006)
Supplement: S4 Table — (DOC) [file pone.0153006.s009.doc]

**S4 Table. The top 200 candidates of three methods on ten function categories.**

|  | **1-50** | | | **51-100** | | | **101-150** | | | **151-200** | | |
| --- | --- | --- | --- | --- | --- | --- | --- | --- | --- | --- | --- | --- |
| **GO Term** | **FIP** | **Top** | **EN** | **FIP** | **Top** | **EN** | **FIP** | **Top** | **EN** | **FIP** | **Top** | **EN** |
| **GO:0009611** | 14/16 | 8/12 | 13/24 | 7/15 | 12/14 | 12/24 | 10/13 | 5/9 | 8/14 | 8/12 | 6/8 | 13/22 |
| **GO:0006954** | 8/10 | 4/8 | 9/18 | 4/10 | 4/5 | 8/18 | 8/9 | 3/6 | 7/12 | 6/8 | 2/5 | 8/15 |
| **GO:0006955** | 4/5 | 4/7 | 8/16 | 2/8 | 0/4 | 8/19 | 8/9 | 3/7 | 8/16 | 8/8 | 2/5 | 9/27 |
| **GO:0032101** | 9/9 | 3/4 | 5/5 | 5/7 | 3/4 | 2/6 | 4/4 | 3/3 | 3/6 | 6/8 | 1/2 | 3/5 |
| **GO:0032496** | 9/10 | 0/1 | 1/2 | 2/7 | 0/0 | 2/4 | 6/6 | 2/4 | 0/2 | 3/4 | 1/2 | 2/2 |
| **GO:0030168** | 8/8 | 1/1 | 0/0 | 2/6 | 0/0 | 1/1 | 5/6 | 1/1 | 1/1 | 4/5 | 0/0 | 3/3 |
| **GO:0042060** | 8/9 | 4/5 | 6/10 | 3/7 | 0/1 | 4/7 | 2/3 | 3/3 | 1/2 | 2/3 | 2/3 | 5/7 |
| **GO:0051241** | 4/5 | 3/3 | 5/24 | 4/4 | 2/3 | 0/3 | 1/3 | 1/3 | 4/6 | 4/6 | 0/0 | 1/3 |
| **GO:0050878** | 7/7 | 3/3 | 6/10 | 0/2 | 0/1 | 3/5 | 3/5 | 3/4 | 1/1 | 2/3 | 1/1 | 4/5 |
| **GO:0007596** | 6/6 | 3/5 | 6/10 | 0/2 | 0/1 | 3/5 | 2/3 | 3/3 | 1/1 | 2/2 | 1/1 | 4/5 |

The numbers in the slash left and right present the number of had confirmed genes a by literature and the number of candidate genes, respectively.
