# Supplementary material for: A Novel Prioritization Method in Identifying Recurrent Venous Thromboembolism-Related Genes
Source: PLoS One. 2016 Apr 6;11(4):e0153006. doi: 10.1371/journal.pone.0153006 (PMC4822849; doi:10.1371/journal.pone.0153006)
Supplement: S5 Table — (DOC) [file pone.0153006.s010.doc]

**S5 Table. The top 200 candidates among three methods at four pathways. .**

| **pathway** | **coagulation cascades** | | | **cell adhesion molecules** | | | **hematopoietic cell lineage** | | | **cytokine-cytokine receptor interaction** | | |
| --- | --- | --- | --- | --- | --- | --- | --- | --- | --- | --- | --- | --- |
| **rank/method** | **FIP** | **ToppNet** | **Endeavour** | **FIP** | **ToppNet** | **Endeavour** | **FIP** | **ToppNet** | **Endeavour** | **FIP** | **ToppNet** | **Endeavour** |
| **1-50** | 6/6 | 2/2 | 3/3 | 3/4 | 0/0 | 1/2 | 1/3 | 0/1 | 2/4 | 3/4 | 2/5 | 3/7 |
| **51-100** | 1/1 | 2/2 | 2/2 | 1/2 | 0/2 | 0/2 | 0/1 | 0/0 | 1/1 | 3/5 | 1/1 | 4/14 |
| **101-150** | 1/1 | 1/1 | 0/0 | 0/0 | 1/1 | 2/4 | 0/0 | 1/4 | 1/6 | 1/2 | 1/5 | 5/12 |
| **151-200** | 1/1 | 1/1 | 2/2 | 1/3 | 3/4 | 2/3 | 2/4 | 0/3 | 1/5 | 5/7 | 1/3 | 4/8 |

The numbers in the slash left and right present the number of had confirmed genes by literature and the number of candidate genes, respectively.
