# Supplementary material for: A Novel Prioritization Method in Identifying Recurrent Venous Thromboembolism-Related Genes
Source: PLoS One. 2016 Apr 6;11(4):e0153006. doi: 10.1371/journal.pone.0153006 (PMC4822849; doi:10.1371/journal.pone.0153006)
Supplement: S6 Table — (DOC) [file pone.0153006.s011.doc]

**S6 Table. The literature validation among four parameter combinations for top 200 candidates.**

| **α=0.8, β=0.5, γ=0.9** |  | **α=0.7, β=0.5, γ=0.8** |  | **α=0.9, β=0.5, γ=0.8** |  | **α=0.9, β=0.6, γ=0.8** |  |
| --- | --- | --- | --- | --- | --- | --- | --- |
| ALPL | PMID: 22672431 | ALPL | PMID: 22672431 | ALPL | PMID: 22672431 | ALPL | PMID: 22672431 |
| FGF2 | PMID: 23012273 | FGF2 | PMID: 23012273 | FGF2 | PMID: 23012273 | FGF2 | PMID: 23012273 |
| TCF7L2 | PMID: 24829485 | ITGA2B | PMID: 26773046 | C5AR1 | PMID: 25830298 | C5AR1 | PMID: 25830298 |
| ITGA2B | PMID: 26773046 | F2R | PMID: 26446588 | ITGA2B | PMID: 26773046 | ITGA2B | PMID: 26773046 |
| F2R | PMID: 26446588 | SELPLG | PMID: 18182036 | F2R | PMID: 26446588 | F2R | PMID: 26446588 |
| SELPLG | PMID: 18182036 | C5AR1 | PMID: 25830298 | TCF7L2 | PMID: 24829485 | SERPINF2 | PMID: 20696792 |
| C5AR1 | PMID: 25830298 | PPARGC1A |  | C3AR1 | PMID: 21526204 | SELPLG | PMID: 18182036 |
| SERPINF2 | PMID: 20696792 | C3AR1 | PMID: 21526204 | PPARGC1A |  | C3AR1 | PMID: 21526204 |
| C3AR1 | PMID: 21526204 | SERPINF2 | PMID: 20696792 | SELPLG | PMID: 18182036 | TCF7L2 | PMID: 24829485 |
| APOA1 | PMID: 22955992 | APOA1 | PMID: 22955992 | SERPINF2 | PMID: 20696792 | KIF23 |  |
| F3 | PMID: 26383585 | F3 | PMID: 26383585 | KIF23 |  | APOA1 | PMID: 22955992 |
| ACADSB |  | KIF23 |  | KIF20A |  | F3 | PMID: 26383585 |
| CD4 | PMID: 25001165 | CD4 | PMID: 25001165 | F3 | PMID: 26383585 | IGF2BP3 |  |
| KIF23 |  | SRC | PMID: 26320263 | IGF2BP3 |  | FN1 |  |
| SRC | PMID: 26320263 | FN1 |  | APOA1 | PMID: 22955992 | KIF20A |  |
| VCAM1 | PMID: 18402813 | SCARB1 | PMID:22652597 | CD4 | PMID: 25001165 | CD4 | PMID: 25001165 |
| SCARB1 | PMID:22652597 | VCAM1 | PMID: 18402813 | FN1 |  | VCAM1 | PMID: 18402813 |
| FN1 |  | REN |  | CSF2RB |  | PLAU | PMID: 25790727 |
| PLAU | PMID: 25790727 | CYP2C19 | PMID: 26147597 | VEGFA | PMID: 25006132 | POLD1 |  |
| REN |  | PLAU | PMID: 25790727 | ACADSB |  | F2RL1 | PMID:12069753 |
| POLD1 |  | CYP2B6 | PMID: 24245489 | PLAU | PMID: 25790727 | F8A1 |  |
| CYP2C19 | PMID: 26147597 | CYP3A43 |  | VCAM1 | PMID: 18402813 | POLE |  |
| CYP2B6 | PMID: 24245489 | AKT1 | PMID: 21821713 | F8A1 |  | VTN | PMID: 23041018 |
| CYP3A43 |  | POLD1 |  | F2RL1 | PMID:12069753 | APOA2 | PMID: 24829485 |
| AKT1 | PMID: 21821713 | EGFR | PMID: 26236616 | SRC | PMID: 26320263 | SRC | PMID: 26320263 |
| EGFR | PMID: 26236616 | TICAM1 |  | POLD1 |  | CSF2RB |  |
| POLE |  | TTR |  | IL10RA | PMID: 19134193 | ACADSB |  |
| VTN | PMID: 23041018 | VTN | PMID: 23041018 | AKT1 | PMID: 21821713 | VEGFA | PMID: 25006132 |
| KIF20A |  | APOA2 | PMID: 24829485 | POLE |  | SCARB1 | PMID:22652597 |
| TTR |  | MMP9 | PMID: 23490298 | VTN | PMID: 23041018 | TTR |  |
| F2RL1 | PMID:12069753 | POLE |  | REN |  | F10 | PMID: 22008904 |
| PRL | PMID: 7697624 | PRL | PMID: 7697624 | JUN |  | CYP2C19 | PMID: 26147597 |
| APOA2 | PMID: 24829485 | KIF20A |  | MMP9 | PMID: 23490298 | AKT1 | PMID: 21821713 |
| MMP9 | PMID: 23490298 | STAT3 | PMID: 24736319 | IL9R |  | PRL | PMID: 7697624 |
| CYP2J2 | PMID:17533030 | VEGFA | PMID: 25006132 | PRL | PMID: 7697624 | REN |  |
| MMP2 | PMID: 23490298 | CYP2J2 | PMID:17533030 | UBC |  | CYP2B6 | PMID: 24245489 |
| F10 | PMID: 22008904 | CSF2RB |  | STAT3 | PMID: 24736319 | APOH | PMID: 25081279 |
| APOH | PMID: 25081279 | MMP2 | PMID: 23490298 | CYP2C19 | PMID: 26147597 | A2M | PMID: 20156641 |
| STAT3 | PMID: 24736319 | F2RL1 | PMID:12069753 | CYP2B6 | PMID: 24245489 | CYP3A43 |  |
| ITGAM |  | F10 | PMID: 22008904 | F10 | PMID: 22008904 | MMP9 | PMID: 23490298 |
| A2M | PMID: 20156641 | APOH | PMID: 25081279 | POLA1 |  | IL10RA | PMID: 19134193 |
| JUN |  | A2M | PMID: 20156641 | ITGAM |  | EGFR | PMID: 26236616 |
| TICAM1 |  | PPARA | PMID: 21226266 | CYP3A43 |  | CYP2J2 | PMID:17533030 |
| ELN |  | IL10RA | PMID: 19134193 | APOA2 | PMID: 24829485 | ITGAM |  |
| VEGFA | PMID: 25006132 | JUN |  | TICAM1 |  | POLA1 |  |
| HPX | PMID: 24373343 | MPO | PMID: 23818485 | A2M | PMID: 20156641 | HPX | PMID: 24373343 |
| EDN1 | PMID: 23476046 | ITGAM |  | CD36 |  | JUN |  |
| MPO | PMID: 23818485 | HPX | PMID: 24373343 | EGFR | PMID: 26236616 | STAT3 | PMID: 24736319 |
| CSF2RB |  | EDN1 | PMID: 23476046 | TTR |  | TICAM1 |  |
| CSN1S1 |  | ELN |  | SCARB1 | PMID:22652597 | MPO | PMID: 23818485 |
| SHBG | PMID: 26700933 | EDC3 |  | KLF2 | PMID: 25039491 | EDN1 | PMID: 23476046 |
| EDC3 |  | CSN1S1 |  | TGFBI |  | ELN |  |
| IL10RA | PMID: 19134193 | SHBG | PMID: 26700933 | CYP2J2 | PMID:17533030 | IL9R |  |
| PPARA | PMID: 21226266 | IGF2BP3 |  | MPO | PMID: 23818485 | CSN1S1 |  |
| POLA1 |  | IL9R |  | APOH | PMID: 25081279 | MMP2 | PMID: 23490298 |
| POMC |  | POMC |  | TLR4 | PMID: 26571395 | EDC3 |  |
| THBS1 | PMID: 25343959 | KLF2 | PMID: 25039491 | PPARA | PMID: 21226266 | PPARA | PMID: 21226266 |
| SST | PMID: 25644017 | MAPK1 | PMID: 18471985 | POMC |  | SHBG | PMID: 26700933 |
| MAPK3 | PMID: 17332680 | THBS1 | PMID: 25343959 | EDC3 |  | POMC |  |
| KLF2 | PMID: 25039491 | MAPK3 | PMID: 17332680 | MMP2 | PMID: 23490298 | KLF2 | PMID: 25039491 |
| GOT2 |  | RELA |  | EDN1 | PMID: 23476046 | THBS1 | PMID: 25343959 |
| MMP14 | PMID: 16171603 | SST | PMID: 25644017 | MAPK1 | PMID: 18471985 | UBC |  |
| MAPK1 | PMID: 18471985 | PLA2G2A |  | HPX | PMID: 24373343 | SST | PMID: 25644017 |
| CFTR | PMID: 23506284 | SELL |  | CSN1S1 |  | APP | PMID: 17172227 |
| IGF2BP3 |  | GSTA1 |  | ELN |  | APCS |  |
| PDE4A |  | PDE4A |  | FOS | PMID: 15201277 | GOT2 |  |
| PLA2G2A |  | POLA1 |  | APP | PMID: 17172227 | CFI | PMID: 23555663 |
| RELA |  | GOT2 |  | IFNG | PMID: 26690514 | SELL |  |
| GSTA1 |  | UBC |  | SELL |  | APOC1 | PMID: 23579966 |
| SELL |  | TLR4 | PMID: 26571395 | THBS1 | PMID: 25343959 | TLR4 | PMID: 26571395 |
| ERBB2 | PMID: 26221378 | ERBB2 | PMID: 26221378 | IL13RA1 |  | AFP | PMID: 26576554 |
| IL9R |  | SOCS3 | PMID: 19132239 | GOT2 |  | TGFBI |  |
| LCAT | PMID: 6399344 | CFTR | PMID: 23506284 | FTH1 |  | SERPINA1 | PMID: 23188791 |
| DCN | PMID: 24947404 | APOBEC3G |  | SST | PMID: 25644017 | FTH1 |  |
| HBEGF | PMID: 22402363 | GPX5 |  | SHBG | PMID: 26700933 | RELA |  |
| CD79A | PMID: 17317545 | MMP14 | PMID: 16171603 | SOCS3 | PMID: 19132239 | CD36 |  |
| TNFRSF1B |  | TNFRSF1B |  | RELA |  | MAPK1 | PMID: 18471985 |
| GPX5 |  | LCAT | PMID: 6399344 | APOBEC3G |  | F2RL2 | PMID: 24349080 |
| APCS |  | APP | PMID: 17172227 | ITGB2 | PMID: 26188538 | ITGB2 | PMID: 26188538 |
| RAC3 |  | FTH1 |  | IL8 |  | IGFBP1 |  |
| SOCS3 | PMID: 19132239 | SERPINA1 | PMID: 23188791 | F2RL2 | PMID: 24349080 | CD79A | PMID: 17317545 |
| NPY | PMID: 18054939 | ITGB2 | PMID: 26188538 | TNFRSF1A | PMID: 20671416 | SOCS3 | PMID: 19132239 |
| IGFBP1 |  | GPX1 | PMID: 23426106 | TLR2 | PMID: 26476743 | APOBEC3G |  |
| CD40LG | PMID: 25908768 | APOC1 | PMID: 23579966 | PLAUR | PMID: 19552680 | MMP14 | PMID: 16171603 |
| FTH1 |  | NPY | PMID: 18054939 | SERPINA1 | PMID: 23188791 | PLA2G2A |  |
| APOC1 | PMID: 23579966 | CYP2E1 |  | CFI | PMID: 23555663 | ERBB2 | PMID: 26221378 |
| SERPINA1 | PMID: 23188791 | HBEGF | PMID: 22402363 | CTGF | PMID: 25462173 | FOS | PMID: 15201277 |
| APP | PMID: 17172227 | DCN | PMID: 24947404 | APCS |  | LCAT | PMID: 6399344 |
| PTGS1 | PMID: 26559689 | CD79A | PMID: 17317545 | CD79A | PMID: 17317545 | IFNG | PMID: 26690514 |
| ITGB2 | PMID: 26188538 | APCS |  | ERBB2 | PMID: 26221378 | MBL2 | PMID: 25482922 |
| KDR | PMID: 22626841 | IGFBP1 |  | STAT6 | PMID: 25767272 | CFTR | PMID: 23506284 |
| GPX1 | PMID: 23426106 | FOS | PMID: 15201277 | IGFBP1 |  | IL13RA1 |  |
| CALR | PMID: 25761617 | TNFRSF1A | PMID: 20671416 | MAPK3 | PMID: 17332680 | PLAUR | PMID: 19552680 |
| FURIN | PMID: 16493485 | CYP2D6 | PMID: 23579966 | CD40LG | PMID: 25908768 | HBEGF | PMID: 22402363 |
| UBC |  | PTGS1 | PMID: 26559689 | MMP14 | PMID: 16171603 | DCN | PMID: 24947404 |
| NFKB1 | PMID: 19095643 | RAC3 |  | TNFRSF1B |  | MAPK3 | PMID: 17332680 |
| CFI | PMID: 23555663 | FURIN | PMID: 16493485 | DCN | PMID: 24947404 | NPY | PMID: 18054939 |
| MBL2 | PMID: 25482922 | ALOX5 | PMID: 24366255 | AFP | PMID: 26576554 | CD40LG | PMID: 25908768 |
| CYP2E1 |  | NFKB1 | PMID: 19095643 | PLA2G2A |  | PDE4A |  |
| EEF1A2 | PMID: 11412682 | CALR | PMID: 25761617 | MBL2 | PMID: 25482922 | ITIH4 |  |
| TNFRSF1A | PMID: 20671416 | PLAUR | PMID: 19552680 | APOC1 | PMID: 23579966 | TNFRSF1B |  |
| G6PD | PMID: 24134175 | MBL2 | PMID: 25482922 | CFTR | PMID: 23506284 | RNASE1 | PMID: 23328880 |
| APOBEC3G |  | UCP1 | PMID: 16844662 | CD44 |  | MTTP |  |
| ALOX5 | PMID: 24366255 | TGFBI |  | NPY | PMID: 18054939 | GPX1 | PMID: 23426106 |
| TLR4 | PMID: 26571395 | STAT6 | PMID: 25767272 | PPBP |  | TLR2 | PMID: 26476743 |
| AVP |  | EEF1A2 | PMID: 11412682 | HBEGF | PMID: 22402363 | TNFRSF1A | PMID: 20671416 |
| MAPK8 |  | IL13RA1 |  | PDE4A |  | CTGF | PMID: 25462173 |
| AR | PMID: 22005299 | CD40LG | PMID: 25908768 | GPX1 | PMID: 23426106 | IL8 |  |
| STAT6 | PMID: 25767272 | CFI | PMID: 23555663 | ALOX5 | PMID: 24366255 | AGXT |  |
| FOS | PMID: 15201277 | TP53 | PMID: 26565403 | GSTA1 |  | FURIN | PMID: 16493485 |
| CYP2D6 | PMID: 23579966 | KDR | PMID: 22626841 | LPAR2 |  | HPR |  |
| PLAUR | PMID: 19552680 | TLR2 | PMID: 26476743 | ACTG1 |  | ABCG5 | PMID: 24166850 |
| RNASE1 | PMID: 23328880 | AVP |  | HPR |  | STAT6 | PMID: 25767272 |
| OSM | PMID: 11460521 | AR | PMID: 22005299 | RAC3 |  | GSTA1 |  |
| PTGIR | PMID: 24557578 | G6PD | PMID: 24134175 | CXCL1 |  | CALR | PMID: 25761617 |
| TIMP2 | PMID: 22344262 | MAPK8 |  | OSM | PMID: 11460521 | RAC3 |  |
| F2RL2 | PMID: 24349080 | PTGIR | PMID: 24557578 | LDLRAP1 |  | GPX5 |  |
| TP53 | PMID: 26565403 | OSM | PMID: 11460521 | FURIN | PMID: 16493485 | PTGS1 | PMID: 26559689 |
| TGFBI |  | F2RL2 | PMID: 24349080 | PTGS1 | PMID: 26559689 | G6PD | PMID: 24134175 |
| MMP12 | PMID: 15845912 | CYP4A11 | PMID: 17405690 | CALR | PMID: 25761617 | MMP12 | PMID: 15845912 |
| AFP | PMID: 26576554 | UCP2 | PMID: 18839467 | RNASE1 | PMID: 23328880 | F2RL3 |  |
| HLA-C | PMID: 1810703 | MMP12 | PMID: 15845912 | TP53 | PMID: 26565403 | KDR | PMID: 22626841 |
| TG | PMID: 22955992 | CYP11B2 | PMID: 21228735 | HLA-B | PMID: 21930318 | LDLRAP1 |  |
| CRH |  | HLA-C | PMID: 1810703 | MAPK14 |  | AVP |  |
| AGER | PMID: 24203067 | AFP | PMID: 26576554 | MMP1 | PMID: 23814055 | EEF1A2 | PMID: 11412682 |
| SERPINA6 |  | SERPINA6 |  | GPX5 |  | LPAR2 |  |
| ACTA2 | PMID: 22946110 | LIPE |  | G6PD | PMID: 24134175 | ITIH2 |  |
| MMP1 | PMID: 23814055 | RNASE1 | PMID: 23328880 | MYD88 | PMID: 25738377 | PPBP |  |
| FGFR2 |  | TIMP2 | PMID: 22344262 | MMP12 | PMID: 15845912 | BHMT | PMID: 22192524 |
| LPAR2 |  | CRH |  | MAPK8 |  | TG | PMID: 22955992 |
| CYP11B2 | PMID: 21228735 | TG | PMID: 22955992 | KDR | PMID: 22626841 | CLU | PMID: 24758255 |
| RAC1 | PMID: 25628054 | AGER | PMID: 24203067 | AVP |  | NFKB1 | PMID: 19095643 |
| CASR |  | F2RL3 |  | RAC1 | PMID: 25628054 | MASP2 | PMID: 25533914 |
| F2RL3 |  | CLU | PMID: 24758255 | F2RL3 |  | ALOX5 | PMID: 24366255 |
| CLU | PMID: 24758255 | GSTA4 |  | CLU | PMID: 24758255 | MMP1 | PMID: 23814055 |
| IL13RA1 |  | ABCG5 | PMID: 24166850 | DHX40 |  | AR | PMID: 22005299 |
| ABCG5 | PMID: 24166850 | FGFR2 |  | LCAT | PMID: 6399344 | OSM | PMID: 11460521 |
| MAS1 |  | ACTA2 | PMID: 22946110 | HLA-C | PMID: 1810703 | FETUB |  |
| HLA-B | PMID: 21930318 | MAPK14 |  | BHMT | PMID: 22192524 | TIMP2 | PMID: 22344262 |
| UCP2 | PMID: 18839467 | RAC1 | PMID: 25628054 | SYK | PMID: 26518435 | RAC1 | PMID: 25628054 |
| AGXT |  | CASR |  | TIMP2 | PMID: 22344262 | OTC | PMID: 19343772 |
| TLR2 | PMID: 26476743 | SYK | PMID: 26518435 | ITIH4 |  | KLKB1 | PMID: 25684211 |
| SYK | PMID: 26518435 | HLA-B | PMID: 21930318 | NFKB1 | PMID: 19095643 | PTGIR | PMID: 24557578 |
| GFAP | PMID: 25043249 | MYD88 | PMID: 25738377 | AR | PMID: 22005299 | SYK | PMID: 26518435 |
| CTSB |  | AGXT |  | ACTB |  | ACTA2 | PMID: 22946110 |
| JAK3 | PMID: 15180539 | FASN | PMID: 22675511 | ABCG5 | PMID: 24166850 | MAPK8 |  |
| GSTA4 |  | HNF1A | PMID: 12911579 | JAK3 | PMID: 15180539 | LIPE |  |
| HNF1A | PMID: 12911579 | ABCB11 |  | MTTP |  | APOF |  |
| FASN | PMID: 22675511 | CTSB |  | HP |  | ABCB11 |  |
| MTTP |  | SULT2B1 |  | CXCR4 |  | CD44 |  |
| LIPE |  | SLC10A1 |  | TG | PMID: 22955992 | HP |  |
| CTSD | PMID: 19910633 | GFAP | PMID: 25043249 | AGXT |  | IL5 | PMID: 24178511 |
| MGP | PMID: 26040031 | EPHX1 |  | HSD17B12 |  | SLC10A1 |  |
| CCK | PMID: 11330425 | MTTP |  | IL6R | PMID: 22421340 | SERPINA7 |  |
| IFNG | PMID: 26690514 | CD36 |  | IL5 | PMID: 24178511 | GP1BA | PMID: 22872156 |
| SULT2B1 |  | JAK3 | PMID: 15180539 | IL2RA |  | FGFR2 |  |
| LDLRAP1 |  | MMP1 | PMID: 23814055 | ABCA1 |  | FASN | PMID: 22675511 |
| CDH5 |  | OTC | PMID: 19343772 | HGF |  | CYP2E1 |  |
| OTC | PMID: 19343772 | CTGF | PMID: 25462173 | CCR1 |  | JAK3 | PMID: 15180539 |
| TH |  | PPBP |  | PTGIR | PMID: 24557578 | HLA-B | PMID: 21930318 |
| CTGF | PMID: 25462173 | MAS1 |  | GSTA4 |  | MAS1 |  |
| ENG |  | DRD2 |  | CCR3 |  | TP53 | PMID: 26565403 |
| ABCB11 |  | RXRA |  | EEF1A2 | PMID: 11412682 | CXCL1 |  |
| MASP2 | PMID: 25533914 | MGP | PMID: 26040031 | PLA2G7 |  | EPHX1 |  |
| SLC10A1 |  | HPR |  | CLEC3B |  | UCP2 | PMID: 18839467 |
| MBP | PMID: 20888633 | MASP2 | PMID: 25533914 | ACTA2 | PMID: 22946110 | SAA4 |  |
| HPR |  | IFNG | PMID: 26690514 | CREBBP |  | CRH |  |
| CDH2 |  | CCK | PMID: 11330425 | FASN | PMID: 22675511 | CTSB |  |
| CYP4A11 | PMID: 17405690 | ADRBK1 |  | UCP2 | PMID: 18839467 | CDH5 |  |
| CD80 | PMID: 12682232 | CDH5 |  | LYN |  | SERPINA6 |  |
| EPHX1 |  | CTSD | PMID: 19910633 | CTSB |  | HLA-C | PMID: 1810703 |
| GP1BA | PMID: 22872156 | TH |  | IL18 |  | HNF1A | PMID: 12911579 |
| ITIH4 |  | GPX4 | PMID: 23770613 | TYK2 |  | GH1 | PMID: 21273694 |
| MYD88 | PMID: 25738377 | LPAR2 |  | MGP | PMID: 26040031 | CLEC3B |  |
| CMA1 |  | GP1BA | PMID: 22872156 | GP1BA | PMID: 22872156 | MGP | PMID: 26040031 |
| BHMT | PMID: 22192524 | VEGFC | PMID: 16621967 | KLKB1 | PMID: 25684211 | MAPK14 |  |
| CCL17 | PMID: 18723831 | PIK3CD |  | CRH |  | GSTA4 |  |
| ADRBK1 |  | FETUB |  | FGFR2 |  | SLC27A5 |  |
| GPX4 | PMID: 23770613 | ITIH4 |  | PIK3CD |  | APOM |  |
| DRD2 |  | MBP | PMID: 20888633 | RXRA |  | AGER | PMID: 24203067 |
| FETUB |  | CD80 | PMID: 12682232 | ABCB11 |  | CCK | PMID: 11330425 |
| MAPK14 |  | CCL17 | PMID: 18723831 | HNF1A | PMID: 12911579 | CCL17 | PMID: 18723831 |
| IL5 | PMID: 24178511 | IL6R | PMID: 22421340 | ITIH2 |  | GFAP | PMID: 25043249 |
| GAST |  | KLKB1 | PMID: 25684211 | CASR |  | CMA1 |  |
| GH1 | PMID: 21273694 | IL5 | PMID: 24178511 | MAS1 |  | GPX4 | PMID: 23770613 |
| TNNT2 |  | CDH2 |  | LIPE |  | CCR3 |  |
| BGN |  | CMA1 |  | MASP2 | PMID: 25533914 | VEGFC | PMID: 16621967 |
| CD19 | PMID: 21571767 | CLEC3B |  | CCL17 | PMID: 18723831 | ENG |  |
| KLKB1 | PMID: 25684211 | ENG |  | OTC | PMID: 19343772 | RNASE2 |  |
| VEGFC | PMID: 16621967 | ATP4A |  | CXCL10 |  | RETN |  |
| CX3CL1 | PMID: 22244861 | FABP4 |  | FETUB |  | CASR |  |
| CLEC3B |  | IL8 |  | RETN |  | MYD88 | PMID: 25738377 |
| RXRA |  | SREBF2 |  | EPHX1 |  | FABP4 |  |
| RNASE2 |  | RETN |  | MBP | PMID: 20888633 | IL6R | PMID: 22421340 |
| TNC |  | BGN |  | KIAA0101 |  | TNC |  |
| UCP1 | PMID: 16844662 | CX3CL1 | PMID: 22244861 | VEGFC | PMID: 16621967 | ANGPTL3 |  |
| IL6R | PMID: 22421340 | ITIH2 |  | CCK | PMID: 11330425 | CCR1 |  |
| PIK3CD |  | HBA2 |  | GFAP | PMID: 25043249 | PNLIP |  |
| ITIH2 |  | RNASE2 |  | SLC10A1 |  | CYP4A11 | PMID: 17405690 |
| PTPN1 | PMID: 25042561 | LPO |  | CDH5 |  | CTSD | PMID: 19910633 |
